# UPF3B modulates endoplasmic reticulum stress through interaction with inositol-requiring enzyme-1α

**DOI:** 10.1038/s41419-024-06973-3

**Published:** 2024-08-13

**Authors:** XingSheng Sun, Ruqin Lin, Xinxia Lu, Zhikai Wu, Xueying Qi, Tianqing Jiang, Jun Jiang, Peiqiang Mu, Qingmei Chen, Jikai Wen, Yiqun Deng

**Affiliations:** 1 State Key Laboratory of Swine and Poultry Breeding Industry, South China Agricultural University, Guangzhou, 510642 Guangdong China; 2 Guangdong Academy of Agricultural Sciences, Guangzhou, 510640 Guangdong China; 3 Guangdong provincial key laboratory for the development biology and environmental adaptation of agricultural organisms, South China Agricultural University, Guangzhou, 510642 Guangdong China

**Keywords:** Endoplasmic reticulum, Stress signalling

## Abstract

The unfolded protein response (UPR) is a conserved and adaptive intracellular pathway that relieves the endoplasmic reticulum (ER) stress by activating ER transmembrane stress sensors. As a consequence of ER stress, the inhibition of nonsense-mediated mRNA decay (NMD) is due to an increase in the phosphorylation of eIF2α, which has the effect of inhibiting translation. However, the role of NMD in maintaining ER homeostasis remains unclear. In this study, we found that the three NMD factors, up-frameshift (UPF)1, UPF2, or UPF3B, were required to negate the UPR. Among these three NMD factors, only UPF3B interacted with inositol-requiring enzyme-1α (IRE1α). This interaction inhibited the kinase activity of IRE1α, abolished autophosphorylation, and reduced IRE1α clustering for ER stress. BiP and UPF3B jointly control the activation of IRE1α on both sides of the ER membrane. Under stress conditions, the phosphorylation of UPF3B was increased and the phosphorylated sites were identified. Both the UPF3B^Y160D^ genetic mutation and phosphorylation at Thr169 of UPF3B abolished its interaction with IRE1α and UPF2, respectively, leading to activation of ER stress and NMD dysfunction. Our study reveals a key physiological role for UPF3B in the reciprocal regulatory relationship between NMD and ER stress.

## Introduction

The endoplasmic reticulum (ER) is a cellular organelle consisting of a system of membranes that is the site of protein and lipid synthesis and regulates intracellular protein folding and trafficking [1]. External stimuli disrupt the function of homeostatic factors in the ER, resulting to the disruption of protein synthesis and the accumulation of unfolded or misfolded proteins, ultimately leading to ER stress [2, 3]. To respond to stress, cells activate an intracellular signaling pathway called the unfolded protein response (UPR) [4, 5]. The UPR is a highly conserved cellular process in all eukaryotes that enhances the protein folding and processing capabilities of the ER and restores ER homeostasis [2, 3, 6]. The UPR consists of three signaling pathways in mammalian cells, including the PERK-eIF2α, IRE1-XBP1, and ATF6 pathways [7]. The IRE1-XBP1 pathway is the most conserved in eukaryotes [8, 9]. In cells, both newly synthesized and pre-existing proteins are under constant threat of misfolding. The accumulation of misfolded proteins disrupts intracellular homeostasis, leading to pathological conditions and even cell death [10]. Upon ER stress, BiP (also known as GRP78), as an ER-resident master regulator protein, dissociates from three key ER transmembrane stress sensors and activates the UPR [11, 12]. As a result, high levels of UPR proteins regulate the expression and activation of the ER stress-related pro-apoptotic proteins CHOP and caspase-12, and the pro-survival molecules GADD34 and BiP, which ultimately determine whether cells undergo apoptosis or adapt to the stress condition [13–15].

Nonsense-mediated mRNA decay (NMD) is a quality control pathway that degrades transcripts carrying premature translation termination codons [16, 17]. As a conserved translation-coupled mRNA quality control mechanism in eukaryotes [18], NMD is estimated to regulate the stability of approximately 5-10% of normal physiological mRNAs [19, 20]. However, the physiological significance of NMD and NMD factors remains to be elucidated. The most conserved NMD factors are the up-frameshift (UPF) proteins UPF1, UPF2, and UPF3 (UPF3B in mammalian cells). The human NMD machinery consists of additional morphogenetic suppressors of genitalia (SMG) proteins [21, 22]. ER stress has been reported to inhibit NMD, possibly due to high phosphorylation of eIF2α [23]. However, the mechanistic role of NMD, in particular the role of UPF1, UPF2, and UPF3B in ER stress, remains unclear.

In this study, we first demonstrate that knockdown of any of the NMD factors, UPF1, UPF2, and UPF3B, activated ER stress and led to cell apoptosis. Among the three key NMD factors, UPF3B showed a unique regulatory role in inhibiting the activation of IRE1α. UPF3B interacted with IRE1α and bound to its kinase domain. This interaction inhibited the phosphorylation and oligomerization of IRE1α and subsequently attenuated the extent of ER stress. BiP and UPF3B jointly controlled the activation of IRE1α on both sides of the ER membrane. In addition, overexpression of UPF2 competed with UPF3B to inhibit the interaction between IRE1α and UPF3B. UPF3B was apparently phosphorylated under stress stimuli with tunicamycin (Tm) or thapsigargin (Tg) treatment. Phosphorylation of UPF3B at threonine169 and the UPF3B^Y160D^ genetic mutation attenuated the interaction of UPF3B with IRE1α and UPF2, respectively. Overexpression of both mutants, UPF3B^Y169D^ and UPF3B^Y160D^, did not rescue the apoptosis caused by UPF3B depletion. This suggests that the phosphorylation of UPF3B may contribute to the ER stress-induced pathogenesis. In conclusion, our data demonstrate that UPF3B plays a critical role in ER homeostasis by inhibiting the UPR and preventing ER stress-induced cell apoptosis.

## Results

### NMD regulates the UPR signaling pathway

**Fig. 1:**
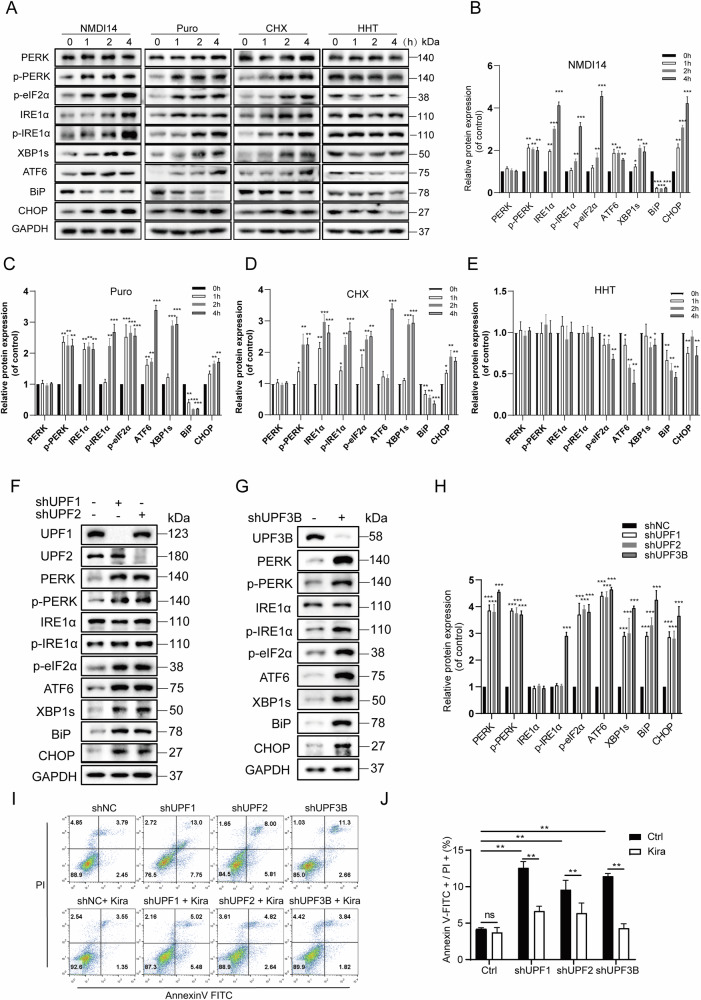
Translation and NMD inhibitors or knock-down of NMD factors activate the UPR signaling pathway. **A**–**E** The expression of ER stress-related proteins was evaluated under incubation with 50 µM NMDI14, 2 µM harringtonine, 100 µg/mL puromycin and 100 µg/mL cycloheximide, respectively, for 1 or 2 or 4 h. The cells were then harvested and subjected to western blotting analysis and quantification (*n* = 3). **F**–**H** UPF1, UPF2, and UPF3B were knocked down stably in HEK293T cells to detect the expression level of target proteins. A negative control is the vector pLKO.1-TRC containing non-hairpin insert. All the protein levels were analyzed by western blotting and quantification (*n* = 3). **I** Flow cytometry analysis the apoptosis of shUPF cells with or without Kira6 treatment (50 μM for 6 h). Annexin V labeled with fluorescein FITC was used as a probe to detect the occurrence of apoptosis by flow cytometry. Annexin-V, a Ca^2+^-dependent phospholipid-binding protein that binds to phosphotidylserine with high affinity. **J** Data statistical analyses were performed on Annexin-V+/PI+ double-positive cells in **I**. Image **J** software was used for the grayscale calculations, and then the data were statistically analyzed for significance. The results are the means ± SEMs of at three independent experiments. Statistical significance was defined as *p < 0.05, **p < 0.01, ***p < 0.001.

NMD is the translation-coupled mRNA degradation pathway that functionally eliminates the overproduction of truncated proteins both in the ER and in the cytosol. To test whether inhibition of NMD affects ER stress, the NMD inhibitor NMDI14 and several protein synthesis inhibitors with different mechanisms of translation inhibition were selected for treatment of HEK293T cells. NMDI14, disrupts the SMG7-UPF1 interactions and thus inhibits NMD [24]. Puromycin, an aminoacyl-tRNA analogue, causes premature termination of translation and leads to rapid polysome degradation [25]. Cycloheximide binds to 80 S ribosomes and prevents tRNA translocation during translation [26, 27]. The cell counting kit-8 (CCK-8) assay was used to demonstrate the effect of the inhibitors on cell survival. The results showed no significant changes in cell death when the inhibitors were treated at different times (Fig. S1). Both translational and NMD inhibitors activated the phosphorylation of eIF2α, PERK, and IRE1α and increased the protein levels of XBP1s, ATF6, and CHOP. However, harringtonine, which only blocks translational elongation without inhibiting NMD [28], neither caused the increase in eIF2α phosphorylation nor led to ER stress (Fig. 1A–E).

Next, three key factors, UPF1, UPF2, and UPF3B, were depleted in HEK293T cells, respectively. In all UPF-depleted cells, phosphorylation of PERK and eIF2α and protein levels of ATF6, XBP1s, BiP, and CHOP were apparently upregulated (Fig. 1F–H), similar to treatment with NMDI14, puromycin or cycloheximide. These data suggest that the maintenance of proper NMD function is critical for balancing the basal activation of the UPR pathway. Several studies have reported that the NMD pathway regulates the UPR [29], and several mRNAs encoding UPR components that are targeted for degradation by NMD. We then investigated the effect of these NMD factors on apoptosis. In all three UPF-depleted cell lines, cell apoptosis was apparently increased (Fig. 1I, J). To determine whether the NMD factor depletion-induced apoptosis was mediated by the activation of ER stress, the IRE1α inhibitor Kira6 was selected to treat the UPF-depleted cells. Kira6 is an imidazopyrazine-based small molecule that competitively binds the ATP-binding site of the kinase domain of IRE1α and blocks the kinase and RNase activities of IRE1α [30, 31]. Cell apoptosis induced by NMD disruption is significantly alleviated by Kira6 treatment. This suggests that the high level of cell apoptosis upon NMD disruption is mediated by activation of ER stress, possibly related to the IRE1α branch pathway.

### Unique regulatory role of UPF3B in IRE1α signaling pathway

**Fig. 2:**
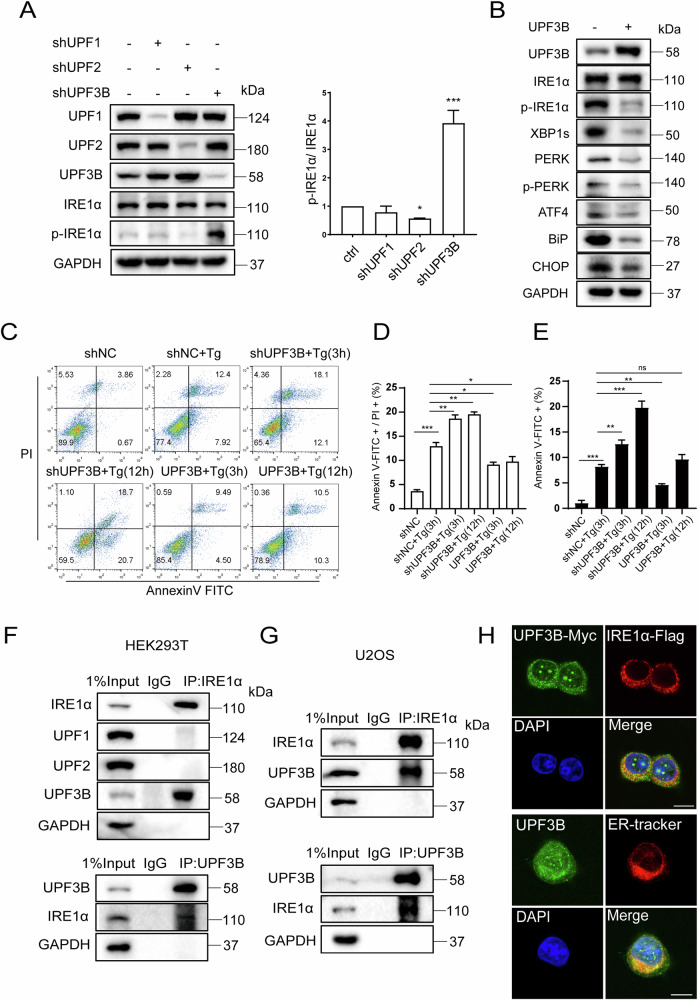
Unique regulation of UPF3B in activating the UPR signaling pathway. **A** The levels of phosphorylated IRE1α were evaluated following treatment with shRNA of UPF1, UPF2 and UPF3B in HEK293T cells, respectively. Negative control is the vector pLKO.1-TRC containing non-hairpin insert. The cells were then harvested and subjected to western blotting analysis, and the relative phosphorylation of IRE1α was statistically analyzed (*n* = 3). *p < 0.05, **p < 0.01, ***p < 0.001. **B** The expression levels of ER stress-related proteins were evaluated in overexpressed UPF3B cells. HEK293T cells were transfected with pCMV or pCMV-UPF3B for 24 h. The cells were then harvested and subjected to western blotting analysis. **C**–**E** Flow cytometry analysis apoptosis of shUPF3B and overexpressed UPF3B cells under Tg (2 μM for 3 h or 12 h) treatments. Data statistical analyses were performed on Annexin-V + /PI+ double positive cells and Annexin-V+ single positive cells (n = 3). UPF3B interacts with IRE1α. Co-IP analysis of the interaction between IRE1α and UPF3B in HEK293T (**F**) and U2OS cells (**G**). The cells lysates were subjected to immunoprecipitation (IP) and western blot analysis with the indicated antibodies, IgG was used as a negative control in IP assay. (**H**) Up: the localization of IRE1α and UPF3B was evaluated by an immunofluorescence assay. U2OS cells were fixed and stained with anti-Flag antibody (red), anti-Myc antibody (green) and DAPI (blue). Down: the localization of UPF3B and ER. U2OS cells were fixed and stained with anti-UPF3B antibody (green), ER tracker (red) and DAPI (blue). Scale bar, 10 μm. The results are the means ± SEMs of at three independent experiments. Statistical significance was defined as *p < 0.05, **p < 0.01 or ***p < 0.001.

Surprisingly, UPF3B has a distinct role in IRE1α phosphorylation compared to UPF1 and UPF2. In shUPF3B cells, IRE1α phosphorylation was apparently increased, but this was not the case in shUPF1 and shUPF2 cells. Instead, IRE1α phosphorylation was slightly further inhibited in shUPF2 cells (Fig. 2A). Knockdown of UPF1 or UPF2 slightly increased the protein level of UPF3B, but depletion of UPF3B had no effect on the levels of UPF1 and UPF2. Instead, the levels of IRE1α phosphorylation and XBP1s, PERK phosphorylation and its downstream factor, activating transcription factor 4 (ATF4), were strongly reduced in overexpressed UPF3B cells. In addition to the ER stress markers, the expression levels of two downstream effectors, BiP and CHOP, were also significantly lower than in normal cells (Fig. 2B).

Since UPF3B knockdown leads to apoptosis, the effects of UPF3B on apoptosis during ER stress were investigated. The ER stress inducer Tg was chosen to treat the cells. Tg is a sesquiterpene lactone that is permeable to cells and induces ER stress by specifically inhibiting the Ca^2+^-ATPase within the ER [32]. The results showed that the percentage of cell death induced by Tg is approximately 12% at 3 h. Knockdown of UPF3B enhanced apoptosis induced by Tg treatment at both 3 h and 12 h, but overexpression of UPF3B inhibited Tg-induced apoptosis even after prolonged treatment with Tg at 12 h (Fig. 2C–E). This suggests that UPF3B is involved in the regulation of apoptosis induced by ER stress.

It is interesting to explore the underlying mechanism by which UPF3B uniquely affects the phosphorylation of IRE1α, rather than UPF1 and UPF2. First, the endogenous interaction between IRE1α and UPF3B was confirmed by co-immunoprecipitation assays in both HEK293T cells and the human osteosarcoma cell line, U2OS cells (Fig. 2F, G). To exclude that the interaction between IRE1α and UPF3B is mediated by RNAs, since both are RNA binding proteins, cell lysates were pretreated with RNase A and IRE1α still immunoprecipitated UPF3B (Fig. S2). This suggests that the interaction occurs in an RNA-independent manner. Immunofluorescence experiments and colocalization analysis showed that UPF3B as a shuttling protein, partially colocalized with IRE1α at ER loci (Fig. 2H and S3). Taken together, these data suggest that UPF3B interacts with IRE1α at the ER and modulates the activation of the IRE1α-XBP1s branch.

To confirm whether the specific role of UPF3B in modulating IRE1α is UPF1 and UPF2 dependent or not, the interaction of UPF3B and IRE1α was examined in shUPF1 and shUPF2 cells by endogenous IP (Fig. S4). The interaction of UPF3B and IRE1α was not strongly affected by UPF1 and UPF2 knockdown. The results show that the effect of UPF3B-IRE1α interaction is likely to be independent of UPF1 or UPF2. The expression levels of IRE1α and UPF3B were retrieved from the human proteome map (HPM) database [33] (Fig. S5). Based on LC-MS/MS, this project adopts high-resolution and high-precision Fourier transform mass spectrometry technology. All mass spectrometry data were obtained in high-high mode on an Orbitrap mass spectrometry analyzer including precursors and HCD-derived fragments. The protein expression levels were based on peptides counted by mass spectrum for each gene and showed as the white-to-red heat map. The data suggested that the protein level of UPF3B is comparable with IRE1α in many human tissues or organs.

In vertebrates, there are two homologues of the yeast UPF3 protein, termed UPF3A and UPF3B [34]. UPF3A has been shown to compensate for the loss of function of UPF3B on NMD substrates [35, 36]. Due to the structural similarity between UPF3A and UPF3B, the interaction of UPF3A with IRE1α was investigated and the role of UPF3A in the phosphorylation of IRE1α was also verified. UPF3A interacts with IRE1α (Fig. S6A). Knockdown of UPF3B upregulated the level of UPF3A [37], but knockdown of UPF3A had a minimal effect on the level of UPF3B and IRE1α phosphorylation (Fig. S6B), much weaker than UPF3B did. Furthermore, when UPF3A was overexpressed in the shUPF3B cell line, the phosphorylation of IRE1α was not decrease visibly (Fig. S6C). These results suggest that UPF3A has a much weaker effect on the IRE1α signaling pathway.

### UPF3B inhibits the phosphorylation of IRE1α by binding to its kinase domain

**Fig. 3:**
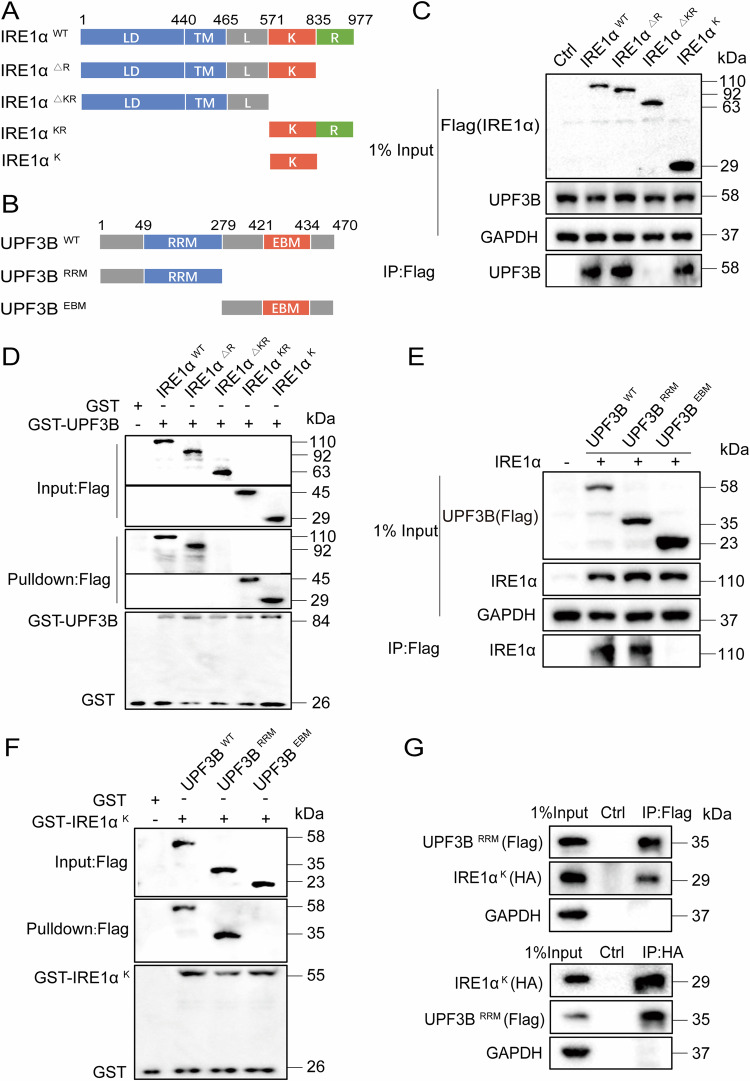
The kinase domain of IRE1α interacts with the RRM domain of UPF3B. **A** Diagram of the wild type (WT) and mutant versions of Flag-tagged IRE1α analyzed. The luminal domain (LD), transmembrane segment (TM), linker region (L), kinase (K), and RNase (R) domains are indicated. **B** Diagram of the WT and mutant versions of Flag-tagged UPF3B analyzed. The RNA recognition motif (RRM) like domain and exon junction complex binding motif (EBM) are indicated. **C** Identification of the IRE1α domain responsible for interacting with UPF3B. HEK293T cells were transfected with IRE1α-Flag deletion mutants. The cell lysates were immunoprecipitated with an anti-Flag antibody, and the precipitates and whole-cell lysates were then analyzed by western blotting. **D** The purified GST or GST-UPF3B fusion protein bound to agarose beads was added to the lysate of HEK293T cells expressing IRE1α-Flag deletion mutants. After GST affinity purification, protein complexes were washed and detected by western blot analysis with anti-Flag or anti-GST as indicated. GST protein was used as a negative control. **E** Identification of the UPF3B domain responsible for interacting with IRE1α. Cells were transfected with IRE1α and the two UPF3B-Flag deletion mutants. The cell lysates were immunoprecipitated with an anti-Flag antibody, and the precipitates and whole-cell lysates were then analyzed by western blotting. **F** The purified GST or GST-IRE1α^K^-fusion protein bound to agarose beads was added to the lysate of HEK293T cells expressing UPF3B-Flag deletion mutants. After GST affinity purification, protein complexes were washed and detected by western blot analysis with anti-Flag or anti-GST as indicated. GST protein was used as a negative control. **G** The IRE1α kinase domain was interaction with UPF3B RRM-like region. HEK293T cells were transfected with plasmids encoding the indicated deletion mutants. The cell lysates were immunoprecipitated with anti-Flag antibodies and indicated antibodies, and the precipitates and whole cell lysates were then analyzed by western blotting. The results are from three independent experiments.

IRE1α is a transmembrane protein consisting of a sensory domain in the ER lumen, the transmembrane segments and two cytoplasmic side domains including the regulated kinase domain and the RNase domain [38, 39]. UPF3B contains a conserved RNA recognition motif (RRM)-like domain that mediates interaction with the MIF4G (middle portion of eIF4G) domain of UPF2, a middle domain, and an EJC binding motif (EBM) [40]. To investigate the structural requirements for IRE1α interaction with UPF3B, different domain deletion or truncation mutants for IRE1α (Fig. 3A) and UPF3B (Fig. 3B) were generated and their interaction regions were analyzed by co-immunoprecipitation assays and GST pulldown experiments. Among these IRE1α mutants, IRE1α^ΔKR^, in which the kinase and RNase domains were deleted, did not interact with UPF3B. However, IRE1α^ΔR^ with the RNase domain deleted, IRE1α^KR^ containing the kinase and RNase domains, and IRE1α^K^ containing only the kinase domai006E interacted with UPF3B (Fig. 3C, D). This suggests that the kinase domain of IRE1α is the UPF3B binding site. In the UPF3B mutants, removal of the RRM-like domain abolished the interaction between UPF3B and IRE1α (Fig. 3E, F), suggesting that the RRM-like domain of UPF3B is the key motif for IRE1α and UPF3B interaction. This was further confirmed by two-way immunoprecipitation analysis between UPF3B^RRM^ and IRE1α^K^ in HEK293T cells (Fig. 3G). However, overexpression of UPF3B^RRM^ alone only minimally suppressed IRE1α phosphorylation, in contrast to overexpression of UPF3B^WT^ (Fig. S7), suggesting that the full length of UPF3B is required for the modulation of IRE1α phosphorylation. To further investigate the effect of UPF3B-IRE1α interaction on NMD, the mRNA levels of several NMD substrates were examined in the presence of complementary UPF3B^WT^ or two truncated mutants, UPF3B^RRM^ and UPF3B^EBM^ in the UPF3B knockdown cell line, respectively (Fig. S8). Indeed, UPF3B knockdown upregulated the mRNA levels of NMD substrates, such as ATF3, ATF4, IRE1α, XBP1s, PERK. The complementation UPF3B^WT^ partially reversed the mRNA levels in UPF3B knockdown cells, but the two truncated mutants UPF3B^RRM^ and UPF3B^EBM^ did not.

### UPF3B prefers to bind unphosphorylated IRE1α

**Fig. 4:**
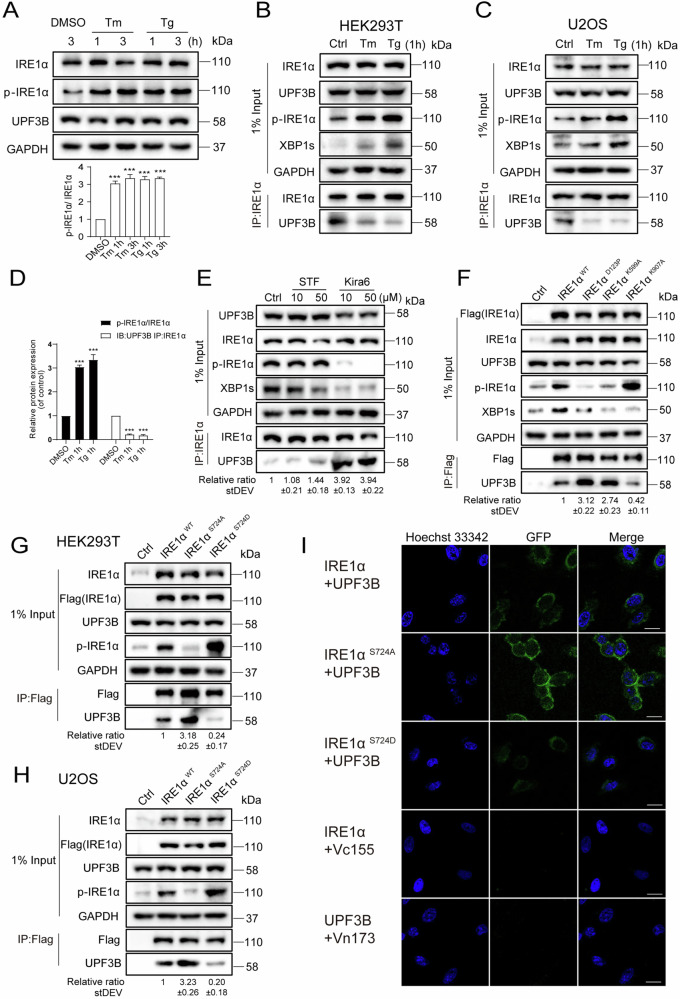
Phosphorylation of IRE1α inhibits its interaction with UPF3B. **A** The levels of phosphorylated IRE1α were evaluated following treatment with dimethyl sulfoxide (DMSO), Tg (2 μM), or Tm (10 μg/mL) for 1 or 3 h. All the target protein levels were analyzed by western blotting and quantification (*n* = 3). **B**–**D** Immunoprecipitation analysis of the endogenous interaction between IRE1α and UPF3B under ER stress in indicated cell lines, HEK293T and U2OS. Cells were incubated with Tg and Tm for 1 h, and then subjected to western blotting analysis and quantification (*n* = 3). The results are the means ± SEMs of at three independent experiments. Statistical significance was defined as *p < 0.05, **p < 0.01 or ***p < 0.001. **E** The effect of kinase and endonuclease activity of IRE1α on the interaction between IRE1α and UPF3B. Cells were incubated with STF and Kira6 (10 or 50 μM) for 6 h. The cell lysates were subjected to immunoprecipitation with the indicated antibodies, and visualized by western blotting. The data statistics of the interaction between IRE1α and UPF3B was showed in Fig. S9. **F** Co-IP analysis of the interaction between IRE1α mutants and UPF3B. HEK293T cells were transfected with the Flag tagged IRE1α mutants for 24 h. The cell lysates were subjected to immunoprecipitation with the indicated antibodies, and visualized by western blotting. pCDNA3.1 transfection was used as a negative control. **G**–**H** Phosphorylation of IRE1α inhibit the interaction between IRE1α and UPF3B in HEK293T and U2OS cell lines. The indicated cell lines were transfected with IRE1α^WT^, the IRE1α^S724^ mutants for 24 h. The cells were then harvested and subjected to western blotting analysis. The data statistics of the interactions were showed in Fig. S9. **I** The co-localization of IRE1α with UPF3B was evaluated by BiFC. IRE1α-Vn173 and UPF3B-Vc155 constructs and the mutants were transfected into U2OS cells, then stained with Hoechst 33342. The figures show representative fluorescent images of the indicated proteins. Scale bar, 20 μm.

Tg and Tm were chosen to treat the cells to investigate whether the interaction between IRE1α and UPF3B is affected under ER stress activation. Tm is a natural nucleoside antibiotic that induces ER stress by inhibiting the protein glycosylation pathway [41]. When cells were treated with Tm (10 μg/mL) or Tg (2 μM) for 1 and 3 h, respectively, the phosphorylation level of IRE1α was significantly upregulated, but the expression level of UPF3B was not affected (Fig. 4A). However, the interaction between IRE1α and UPF3B was significantly decreased in HEK293T and U2OS cells treated with either Tm or Tg, and the strength of the interaction was negatively correlated with the phosphorylation level of IRE1α (Fig. 4B–D). To further investigate whether the interaction was mainly between unphosphorylated IRE1α and UPF3B, two IRE1α inhibitors, STF-083010 and Kira6, were selected to test the interaction. Unlike Kira6, which inhibited IRE1α phosphorylation and kinase activity, STF-083010 forms a selective Schiff’s base with a catalytic lysine in the RNase active site of IRE1α and is a specific inhibitor of IRE1α endonuclease activity rather than kinase activity [42]. Indeed, the phosphorylation of IRE1α did not change significantly and the splicing form of XBP1 was slightly inhibited after STF-083010 treatment in HEK293T cells. The interaction between IRE1α and UPF3B appeared to be slightly increased in 50 μM STF-083010 treated cells (Fig. 4E). In contrast, Kira6 treatment abolished the phosphorylation of IRE1α and the splicing of XBP1, and the interaction between IRE1α and UPF3B was strongly enhanced by 3.9-fold compared with control cells (Fig. 4E). This suggests that UPF3B preferentially binds to the kinase domain of IRE1α in the non-phosphorylated state.

The IRE1α^D123P^ mutation, which abolishes IRE1α dimerization and activation [43], and the IRE1α^K599A^ mutation in the ATP-binding pocket of the kinase domain [44] have been shown to inhibit the IRE1α phosphorylation. In contrast, the IRE1α^K907A^ mutant was RNase defective but caused high phosphorylation of IRE1α [45]. These three functional mutants were used to further investigate the interaction between IRE1α and UPF3B in comparison to IRE1α wild-type (Fig. 4F). Consistent with previous studies, phosphorylation of IRE1α was strongly inhibited in the IRE1α^D123P^ and IRE1α^K599A^ mutants, but enhanced in the IRE1α^K907A^ mutant. More importantly, the interaction was apparently stronger between UPF3B and IRE1α^D123P^ or IRE1α^K599A^, but weaker between UPF3B and IRE1α^K599A^ compared to the wild-type interaction (Fig. 4F). This further confirms that phosphorylation of IRE1α abolished the interaction with UPF3B. Since phosphorylation at Ser724 of IRE1α is the predominant activated form of the kinase, we substituted Ser724 of IRE1α with aspartic acid (designated IRE1α^S724D^) or alanine (IRE1α^S724A^) to mimic the retention or loss of its kinase activity, respectively. UPF3B has a much stronger interaction with IRE1α^S724A^, 3.2-fold higher, but a much weaker interaction with IRE1α^S724D^, compared with IRE1α^WT^ in HEK293T and U2OS cells (Fig. 4G, H). In summary, the strength of the interaction between IRE1α and UPF3B was negatively correlated with the phosphorylation level of IRE1α (Fig. S9). Next, a bimolecular fluorescence complementation (BiFC) assay was performed to confirm the interaction. IRE1α-Vn173, IRE1α^S724A^-Vn173 and IRE1α^S724D^-Vn173 were co-transfected with UPF3B-Vn155 in U2OS cells, respectively. The results show that the interaction was mainly in the cytoplasm, and the interaction was stronger in IRE1α^S724A^-Vn173 and UPF3B-Vn155 than in IRE1α^S724D^-Vn173 (Fig. 4I), confirming the critical role of phosphorylation at Ser724 of IRE1α in the interaction between IRE1α and UPF3B-Vn155.

### BiP and UPF3B jointly control the activation of IRE1α

**Fig. 5:**
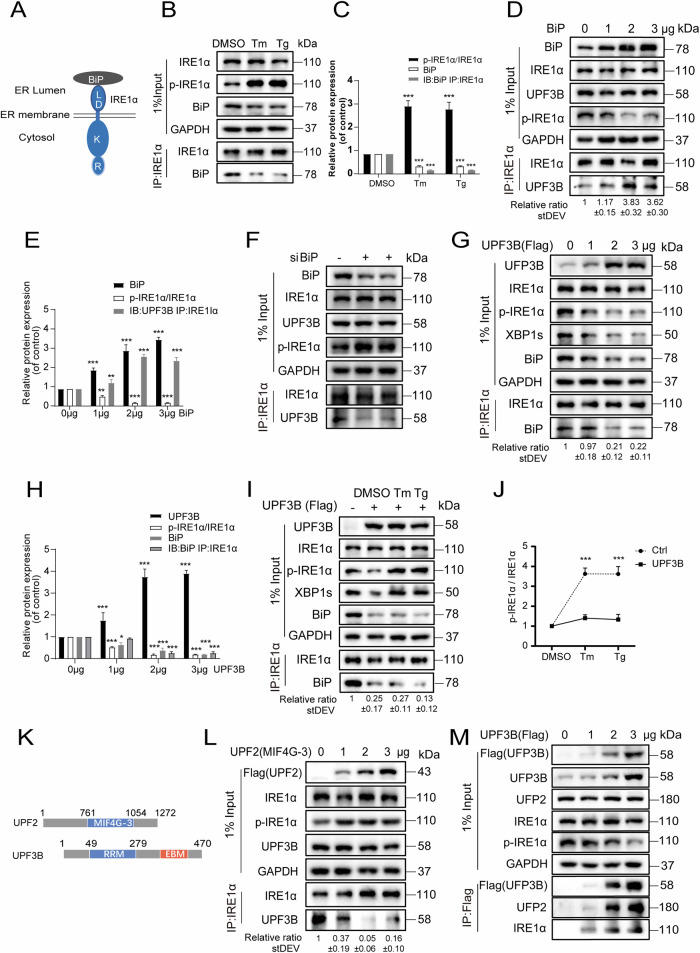
BiP and UPF3B jointly control the activation of IRE1α. **A** Schematic diagram of the ER lumen domain of IRE1α bound to BiP. **B**, **C** The interaction between IRE1α and BiP was inhibited during ER stress. Cells were incubated with Tg and Tm for 1 h, the cell lysates were subjected to immunoprecipitation with the indicated antibodies and visualized by western blotting and quantification (*n* = 3). **D**-**E** BiP enhances the interaction between IRE1α and UPF3B. After 24 h transient overexpressed of BiP plasmid in HEK293T cell line, cells were collected and immunoprecipitation was performed with anti-IRE1α antibody for western blotting analysis and quantification (*n* = 3). **F** siBiP inhibit the interactions of UPF3B and IRE1α. After transfection of 20 nM BiP siRNA or negative control siRNA in HEK293T cell line, cells were collected 48 h later and immunoprecipitation with anti-IRE1α antibody for western blotting analysis. **G**, **H** Overexpressed of UPF3B plasmid in HEK293T cell line, cells were collected and immunoprecipitation with anti-IRE1α antibody for western blotting analysis and quantification (*n* = 3). **I** Overexpressed UPF3B cells were incubated with Tg (2 μM) and Tm (10 μg/mL) for 1 h, respectively, and the cell lysates were subjected to immunoprecipitation with the indicated antibodies, and visualized by western blotting. **J** Overexpressed of UPF3B inhibited IRE1α phosphorylation under ER stress. The data were from Fig. 5B, I. The results are the means ± SEMs of at three independent experiments. Statistical significance was defined as *p < 0.05, **p < 0.01 or ***p < 0.001. **K** Schematic diagram of the structural domains of UPF2 and UPF3B interactions. **L** UPF2 inhibits the interaction between UPF3B and IRE1α by competing with UPF3B. After gradient overexpressed of UPF2 MIF4G-3 plasmid in HEK293T cells for 24 h, cells were collected and the lysates were immunoprecipitated by anti-Flag antibody for western blotting analysis. **M** UPF3B interacts with UPF2 and IRE1α in a dosage-dependent manner. After gradient overexpressed of UPF3B plasmid in HEK293T cells for 24 h, the cell lysates were subjected to immunoprecipitation with the indicated antibodies, and visualized by western blotting.

IRE1α contains four domains, including a sensory domain in the ER lumen, transmembrane segments and two domains in the cytoplasmic side: the regulated kinase domain and the RNase domain (Fig. 5A). BiP has been reported to interact with the sensory domain of IRE1α to attenuate its activation [46]. Under Tm or Tg transient treatment for 1 h, BiP was suppressed, the phosphorylation of IRE1α was increased and accordingly, the interaction between BiP and IRE1α was attenuated (Fig. 5B, C). Restoration of BiP decreased IRE1α phosphorylation and increased the interaction between IRE1α and UPF3B (Fig. 5D, E). In si-BiP cells, the phosphorylation level of IRE1α was increased and the interaction between UPF3B and IRE1α was inhibited (Fig. 5F). These results suggest that BiP in the ER lumen affects the interaction of IRE1α and UPF3B in the cytoplasmic side, possibly via modulation of IRE1α activation.

Overexpression of UPF3B downregulated the levels of phosphorylated IRE1α and BiP, and consequently reduced the interaction between IRE1α and BiP (Fig. 5G, H). This suggests that UPF3B may affect the balance of ER stress by limiting the activation of IRE1α and the expression of BiP. During ER stress activation, the protein level of IRE1α was not affected in UPF3B-overexpressing cells, but the extent of IRE1α phosphorylation and the level of XBP1s were also much less increased compared to the normal cells (Figs. 2B, 5I, J). The interactions between IRE1α and BiP were strongly suppressed due to the downregulation of BiP levels. This may be because UPF3B negates the phosphorylation of IRE1α via protein interactions in the UPR and consequently suppresses the expression of BiP. In shUPF3B cells, phosphorylation of IRE1α was still inhibited by overexpression of BiP (Fig. S10A), but the efficiency of inhibition was only achieved at higher levels of BiP expression than that in normal cells (Fig. S10B). When siBiP was used in UPF3B knockdown cell lines, phosphorylated IRE1α was not further upregulated (Fig. S10C). The results show that UPF3B may have a concerted regulatory role in IRE1α phosphorylation together with BiP, but is not fully dependent on BiP expression. In the BiP knockdown cell lines, overexpression of UPF3B inhibited IRE1α phosphorylation (Fig. S10D), indicating that the functions of UPF3B and BiP in regulating IRE1α activity are independent and redundant.

UPF2 contains three conserved MIF4G (middle part of eIF4G) structural domains [47, 48]. UPF2 interacts with UPF3B through its third MIF4G structural domain (Fig. 5K) and with UPF1 through its C-terminus, forming the central component of the ternary UPF complex. When the UPF2 MIF4G-3 segment was overexpressed in cells (Fig. 5L), the interaction of UPF3B with IRE1α was apparently inhibited and the phosphorylation of IRE1α was increased. Furthermore, overexpression of UPF3B not only decreased the phosphorylation of IRE1α but also increased UPF2 and IRE1α in a dose-dependent manner (Fig. 5M). Two UPF3B mutants, UPF3B^K52E^ or UPF3B^R56E^, which disrupt the interaction of UPF2 with UPF3B [49], were used to test the interaction between UPF3B and UPF2 or IRE1α. Either UPF3B^K52E^ or UPF3B^R56E^ enhanced the interaction between UPF3B and IRE1α, whereas the EJC binding domain deletion mutant (UPF3B^ΔEBD^ with a.a. 421-434 removed) had no such effect (Fig. S11). Nevertheless, these mutants inhibited IRE1α phosphorylation compared to the control, suggesting that free UPF3B, rather than the intact NMD complex, plays an important role in suppressing IRE1α activation.

### UPF3B attenuates IRE1α dimerization and oligomerization under ER stress

**Fig. 6:**
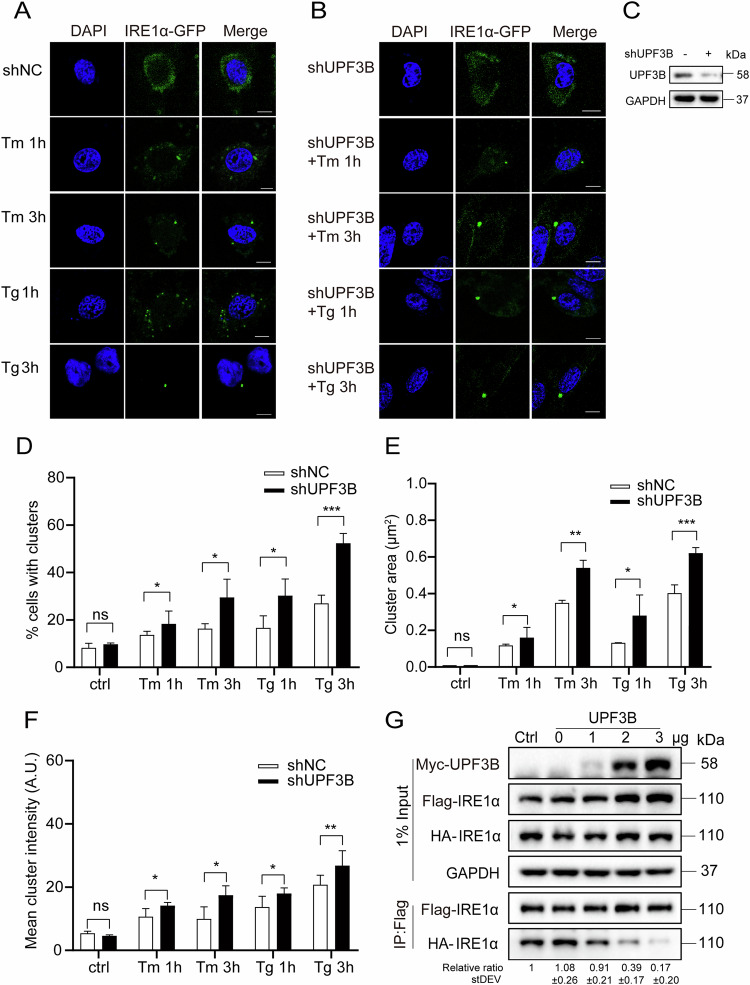
UPF3B inhibits the formation of IRE1α cluster under ER stress. **A**–**C** The IRE1α-GFP plasmid was overexpressed in U2OS cells for 24 h and treated with Tg (2 μM) and Tm (10 μg/mL) for 1 or 3 h in shNC and shUPF3B cells. The cells were stained with DAPI. The scale is 10 μm. Negative control vector containing non-hairpin insert. **D** Percentage of total cell fluorescence intensity found in clusters at each time point. **E** Distribution of cluster sizes during the time course. **F** Distribution of cluster fluorescence intensities at each time point. Each dot represents 1 field, 5 fields were analyzed for each condition and an average of 60 cells were analyzed per condition in each experiment. Image J software was using for data collection. The results are the means ± SEMs of at three independent experiments. Statistical significance was defined as *p < 0.05, **p < 0.01 or ***p < 0.001. **G** UPF3B inhibited IRE1α oligomerization. UPF3B-Myc, IRE1α-Flag, and IRE1α-HA were overexpressed in HEK293T cells. Cells were collected and immunoprecipitation was performed with anti-Flag antibodies for western blotting analysis. pCMV plasmid transfection was used as 0 μg UPF3B control.

During ER stress, activated IRE1α forms higher order oligomers or clusters in stressed cells [50]. Since UPF3B negates IRE1α activation in cells by interaction, it is interesting to confirm whether UPF3B limits the oligomerization of IRE1α during ER stress. IRE1α-GFP was transfected into the control and the shUPF3B cells as an oligomerization indicator. Fluorescent aggregation of IRE1α-GFP was evident under both Tm and Tg treatment for 1 and 3 h, respectively (Fig. 6A), and was further enhanced in shUPF3B cells (Fig. 6B, C). Statistical analysis showed that a higher proportion, larger area and higher fluorescence intensity of aggregated clusters appeared in the shUPF3B cells compared to the control cells (Fig. 6D, F). This suggests that UPF3B is required to prevent the aggregation of IRE1α under ER stress. The activation of IRE1α depends on autophosphorylation induced by its homodimerization, as observed by the changes in the coimmunoprecipitation of the two tagged IRE1α constructs, IRE1α-Flag and IRE1α-HA, which were co-expressed in cells. The dimerization of IRE1α was strongly inhibited with the dose correlating with UPF3B overexpression (Fig. 6G).

### The phosphorylation and genetic mutation of UPF3B abolishes the interaction with IRE1α

**Fig. 7:**
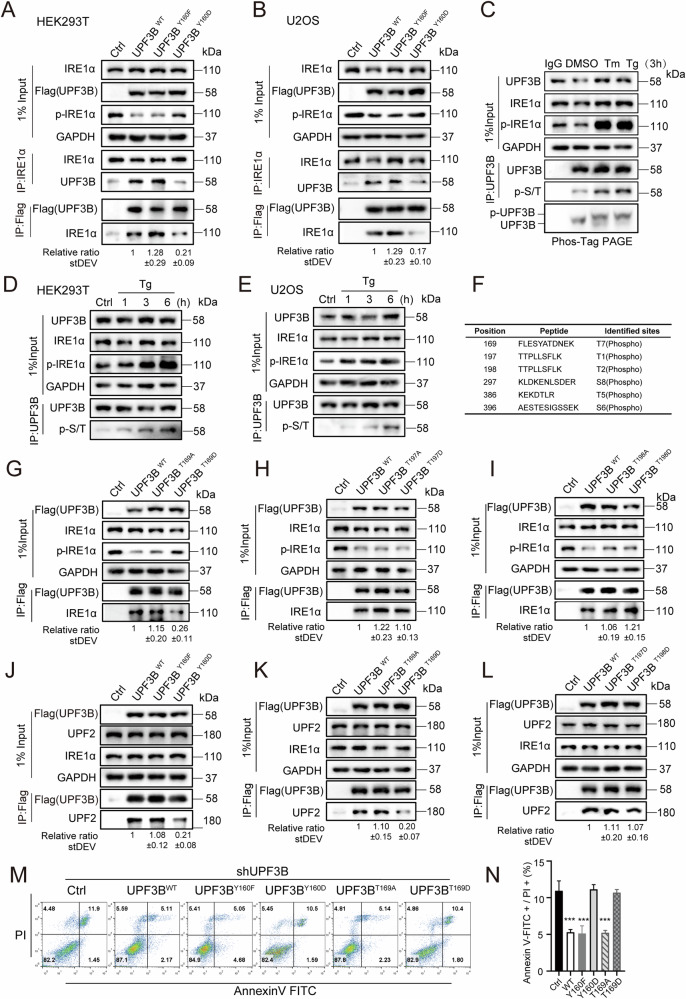
UPF3B is phosphorylated during ER stress. **A**, **B** HEK293T and U2OS cells were transfected with UPF3B^WT^, the UPF3B^Y160F^ mutant, or the UPF3B^Y160D^ mutant for 24 h. Cell lysates were subjected to immunoprecipitation with the Flag or IRE1α antibodies and visualized by western blotting. **C** The expression levels of phosphorylated threonine and serine of UPF3B were evaluated following treatment with Tg (2 μM) and Tm (10 μg/mL) for 3 h. Cell lysates were subjected to immunoprecipitation with the UPF3B antibodies, and visualized by western blotting. **D**, **E** The expression levels of phosphorylated UPF3B were evaluated following treatment with Tg for 1, 3, or 6 h in indicated cell lines. **F** Phosphorylation mapping mass spectrometry of human UPF3B. HEK293T cells were evaluated following treatment with Tg (2 μM) for 6 h. Cell lysates were subjected to immunoprecipitation with the UPF3B antibodies and identification by mass spectrometry. **G** Analysis of the interaction between IRE1α and UPF3B mutants. HEK293T cells were transfected with UPF3B^WT^, the UPF3B^T169A^ mutant, or the UPF3B^T169D^ mutant for 24 h. The cells were harvested for immunoprecipitation with antibodies against Flag. **H**, **I** Cells were transfected with UPF3B mutants for 24 h and then harvested for immunoprecipitation with antibodies against Flag. The data statistics of the interaction was showed in Fig. S13. **J**–**L** Analysis of the interaction between UPF2 and UPF3B mutants. Cells were transfected with UPF3B mutants for 24 h and then harvested for immunoprecipitation with antibodies against Flag. The data statistics of the interaction was showed in Fig. S13. **M**, **N**) Flow cytometry analysis of apoptotic changes supplemented with different UPF3B mutants in shUPF3B cell lines. Data statistical analyses were performed on Annexin-V + /PI+ double positive cells. The results are the means ± SEMs of at three independent experiments. Statistical significance was defined as ***p < 0.001.

A single nucleotide substitution, 478T > G, has been identified in exon 5 of the non-syndromic X-linked mental retardation (XLMR) family [51]. This nucleotide change caused the conversion of tyrosine160 to aspartic acid (Y160D). The tyrosine residue at this site is conserved in UPF3B in plants and animals, suggesting its physiological importance for UPF3B function. However, the underlying pathogenesis remains unknown. We overexpressed two UPF3B mutants, UPF3B^Y160F^ and UPF3B^Y160D^, and immunoprecipitated endogenous IRE1α to detect the interactions between IRE1α and both mutants. UPF3B^Y160D^ showed a weaker interaction with IRE1α compared to UPF3B^WT^ and UPF3B^Y160F^ in HEK293T and U2OS cell lines (Fig. 7A, B), suggesting that UPF3B^Y160D^ loses the function to inhibit IRE1α activation in suppressing ER stress. The oligomerization of IRE1α was examined by complementation of UPF3B^WT^, UPF3B^Y160F^, or UPF3B^Y160D^ in shUPF3B cell lines under ER stress (Fig. S12). Statistical analysis showed that in shUPF3B cells, the proportion, area, and fluorescence intensity of aggregated IRE1α clusters were not suppressed by UPF3B^Y160D^ overexpression, which was similar to control cells, but were apparently suppressed by complementation of UPF3B^WT^ and UPF3B^Y160F^ under ER stress. Taken together, these data suggest that the Y160D mutation may result in chronic high levels of ER stress, which may lead to some neurodevelopmental disorders.

Given that UPF3B^Y160D^ disrupts the interaction between UPF3B and IRE1α, which is similar to the conditions of ER stress activation, we hypothesized that not only IRE1α but also UPF3B is regulated by phosphorylation changes in stress stimuli. First, we examined the changes in serine-threonine phosphorylation of UPF3B in response to ER stress induced by Tm or Tg for 3 h (Fig. 7C). The phosphorylation of UPF3B was increased in a time-dependent manner after 1, 3 or 6 h of Tg treatment, similar to the IRE1α phosphorylation in HEK293T and U2OS cell lines (Fig. 7D, E). The results show that the phosphorylation of UPF3B was increased in response to ER stress. To determine the site where UPF3B was phosphorylated, phosphorylation mapping mass spectrometry was applied to precipitated UPF3B from HEK293T cells (Fig. 7F). Of the six phosphorylation sites identified, T169, T197 or T198 phosphorylation sites were present in the RRM domain that interacts with IRE1α. To address the key phosphorylation sites, we made three pair mutants, UPF3B^T169A^ and UPF3B^T169D^, UPF3B^T197A^ and UPF3B^T197D^, and UPF3B^T198A^ and UPF3B^T198D^, mimicking the unphosphorylated or phosphorylated status of UPF3B. The results show that only UPF3B^T169D^ significantly inhibited the interaction between IRE1α and UPF3B, which is close to the genetic mutation of UPF3B^Y160D^, while the mutations at the other two sites did not significantly alter the interaction (Fig. 7G–I). In conclusion, the strength of the interaction between IRE1α and UPF3B was not only negatively correlated with the phosphorylation level of IRE1α, but also affected by the phosphorylation of UPF3B (Fig. S13).

The interaction of UPF3B^Y160D^ with UPF2 was also inhibited compared to UPF3B^WT^ and UPF3B^Y160F^ (Fig. 7J). Thus, the UPF3B^Y160D^ mutant impaired the interaction of UPF3B with both IRE1α and UPF2. This raises the question of whether XLMR parthenogenesis is due to the loss of the ability of UPF3B^Y160D^ to suppress ER stress maintain NMD efficiency, or both. We therefore investigated whether the potential phosphorylation site of UPF3B would affect its interaction with UPF2. UPF3B^T169D^ also appeared to inhibit the interaction with UPF2 compared to UPF3B^WT^, UPF3B^T197D^ and UPF3B^T198D^ (Fig. 7K, L). The two mutations UPF3B^Y160D^ and UPF3B^T169D^, which were suppressed in the interaction with IRE1α, also attenuated the inhibition of apoptosis compared to the restoration of UPF3B^WT^, UPF3B^Y160F^ or UPF3B^T169A^, respectively, in shUPF3B cell lines under physiological conditions and during ER stress (Figs. 7M, N, S14).

## Discussion

**Fig. 8:**
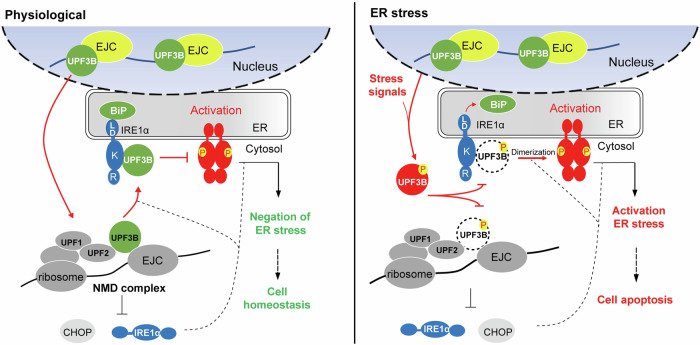
The dual role of UPF3B in NMD and ER stress. Under physiological conditions, UPF3B inhibits the activation of IRE1α and affects its phosphorylation and oligomerization by interacting with the IRE1α kinase domain. UPF3B and BiP jointly control the activation of IRE1α. In addition, NMD can inhibit ER stress by control the expression of IRE1α and CHOP, and negatively feedback ER stress to reshape cell homeostasis. During ER stress, UPF3B is phosphorylated and dissociates from IRE1α, which promotes the expression of IRE1α and CHOP, and activates ER stress, leading to apoptosis.

We discovered the novel function of UPF3B in antagonizing ER stress by specifically inhibiting IRE1α activation under physiological conditions. UPF3B inhibits the formation of higher-order oligomers of IRE1α but not in phosphorylated UPF3B during ER stress (Fig. 8). Our study provides insight into the linkage of two quality control pathways at the ER site through UPF3B interactions. Under physiological conditions, the monomeric IRE1α kinase endonuclease remains in an inactive form by binding to BiP at the sensory domain and UPF3B at the kinase domain on both sides of the ER lumen and cytoplasm. Upon ER stress, unfolded proteins compete with IRE1α for BiP, and stress-induced phosphorylation of UPF3B loses its ability to suppress IRE1α activation. This leads to self-dimerization of IRE1α and mediates its autophosphorylation and IRE1α-dependent decay. The dimerized IRE1α is further oligomerized, and forms foci that correlate with the activation of IRE1α-catalyzed splicing endonuclease activity. UPF3B is phosphorylated during ER stress. UPF3B^T169^ phosphorylation and the UPF3B^Y160D^ genetic mutation fail to interact with IRE1α and UPF2 and are unable to antagonize ER stress-induced apoptosis compared to UPF3B^WT^, which may be related to the pathogenesis of XLMR or other neuronal degenerative diseases. Collectively, our data demonstrate that UPF3B plays an important role in ER homeostasis by inhibiting the UPR and preventing ER stress-induced cell apoptosis.

NMD is a conserved post-transcriptional quality control mechanism in eukaryotes that plays an important role in various physiological and pathological processes such as neurogenesis, synapse formation, nervous system development, and disease [52]. Most NMD studies have focused mainly on the cytosolic mechanism for controlling gene expression. Few studies have examined the physiological function of NMD at ER loci but translation also occurs at the ER. The specific biological functions of NMD in modulating ER stress, beyond the control of gene expression, remain unclear. Understanding whether ER stress is physiologically influenced by NMD is important for our knowledge of the intercellular linkage between different quality control pathways. In our study, depletion of any of the UPF proteins activates ER stress and increases cell apoptosis. When UPF1, UPF2 or UPF3B were depleted in HEK293T cells, phosphorylation of PERK and eIF2α, and protein levels of ATF6, BiP, and CHOP were increased, suggesting that proper levels of NMD factors maintain the suppressive role in ER stress or UPR signaling pathways. It should be noted that ER stress may be indirectly activated when NMD is disrupted, as truncated or unfolded proteins are expected to be highly produced. Thus, efficient NMD may be probably important to prevent inappropriate and prolonged activation of the UPR.

The NMD pathway has been shown to regulate the UPR [29]. NMD directly targets the mRNAs encoding several UPR components, including IRE1α, whose NMD-dependent degradation partly underpins this process. Among the three branches of ER stress, the IRE1α/XBP1 axis is the most conserved pathway. IRE1 is a type I ER transmembrane glycoprotein with Ser/Thr receptor protein kinase activity and specific endonuclease activity [53]. IRE1α is activated to undergo dimerization and autophosphorylation, thereby activating the cytoplasmic endonuclease domain. As a result, the downstream XBP1 mRNA is alternatively spliced and translated into the short protein isoform, XBP1s [54]. XBP1s binds to ER-related cis-transcription elements and upregulates the expression of ER stress-related proteins such as BiP to promote ER membrane biogenesis and enhance the protein folding capacity of the ER to negate further stress [55]. Research has shown that the scaffold protein receptor for activated C-kinase 1 (RACK1) interacts with IRE1α in pancreatic beta cells and primary islets in response to glucose stimulation or ER stress [56]. The ER luminal chaperone ERdj4/DNAJB9 represses IRE1 activation by promoting the complex between BiP and IRE1α at the ER lumen [57]. Li *et al*. found that IRE1α forms a complex with Sec61/Sec63 translocons in cells. Sec63 mediates BiP binding to IRE1α, thereby inhibiting IRE1α oligomerization and attenuating IRE1α signaling during prolonged ER stress [58]. Knockdown of ribosome-associated complex (RAC) has been shown to sensitize mammalian cells to ER stress and selectively interfere with IRE1 branch activation [59]. Higher order oligomerization of the IRE1α kinase/endonuclease is dependent on RAC.

In this study, IRE1α phosphorylation was specifically enhanced by UPF3B knockdown and suppressed by UPF3B overexpression, but not by UPF1 or UPF2 depletion. This suggests that in addition to its role in NMD, UPF3B is specifically involved in the IRE1α/XBP1 axis of the UPR. Overexpression of UPF3B inhibited IRE1α-induced ER stress and cell apoptosis, suggesting a reciprocal role between IRE1α and UPF3B at ER loci in cell fate determination. Indeed, we confirmed that UPF3B interacts with IRE1α by GST pull-down and IP assays, and partially colocalizes with IRE1α by immunofluorescence and BiFC. According to the data from the human proteome map database, the level of UPF3B is comparable with IRE1α, but both proteins amount in HEK293T and U2OS cells remain to be determined. The tight regulatory interplay between these two proteins was investigated in this study. The kinase domain of IRE1α interacts with the RRM-like domain of UPF3B. Under stress conditions, the UPF3B-IRE1α interaction was apparently abolished, mainly due to high phosphorylation of IRE1α. The phosphorylation level of IRE1α is controlled by the phosphorylation of serine at position 724 [60]. The phosphorylation loss mutant IRE1α^S724A^ and IRE1α^WT^ have a higher binding capacity to UPF3B than the phosphorylation mutant IRE1α^S724D^. This confirms that UPF3B has a stronger interaction with non-phosphorylated IRE1α than with phosphorylated IRE1α, suggesting that UPF3B may be involved in the repression of IRE1α phosphorylation. Further analysis of functionally relevant point mutations such as the oligomerization-defective mutation IRE1α^D123P^, the kinase activity-defective mutation IRE1α^K599A^, the RNase activity-defective mutation IRE1α^K907A^ and inhibitors of kinase and RNase activity, supported that UPF3B inhibits IRE1α activation through its interaction with latent IRE1α. We also found that UPF3A, the homolog of UPF3B in human cells, interacts with IRE1α but has a much weaker effect on IRE1α phosphorylation. UPF3A has been suggested to play the redundant role of UPF3B in NMD [37]. However, whether UPF3A has a similar function to UPF3B in the regulation of ER stress remains to be investigated.

BiP acts as the major ER molecular chaperone to inhibit three signaling pathways through luminal interactions. The UPR signaling cascade was maintained at basal levels by BiP. Overexpression of BiP inhibited the phosphorylation of IRE1α and correspondingly increased the interaction between IRE1α and UPF3B. BiP depletion increased the phosphorylation of IRE1α and decreased the interaction between IRE1α and UPF3B. Both suggest that the level of BiP affects the interaction between IRE1α and UPF3B. Interestingly, overexpression of UPF3B not only attenuated ER stress, but also reduced BiP levels. Furthermore, in shUPF3B cells, more BiP expression was required to return IRE1α phosphorylation to baseline, suggesting that UPF3B functions cooperatively and redundantly in suppressing IRE1α activation at the cytoplasmic side. All this confirms that the interaction between BiP and IRE1α on the luminal side potentially interacts with the interaction between UPF3B and IRE1α on the cytoplasmic side.

Phosphorylated IRE1α is prone to dimerization and further oligomerization, which depends on the activation of its cytoplasmic kinase domain. In addition to biochemical evidence of oligomerization, live cell microscopy of IRE1α using fluorescent protein labeling shows that its accumulation in the ER membranes is the hallmark of the extent of ER stress [61]. Using the same strategy, we demonstrated that UPF3B knockdown affected IRE1α clustering. The IP assay showed that the IRE1α oligomerization appeared to be inhibited by UPF3B. These results further confirm that UPF3B suppresses ER stress by inhibiting IRE1α phosphorylation and clustering under stress, such as exposure to Tg and Tm.

UPF3B missense mutations are found in patients with schizophrenia and X-linked intellectual disability (XLID). Expression of these UPF3B mutants in neural stem cells impairs neuronal differentiation and reduces axonal branching [62]. Chronic activation of ER stress also leads to the formation and accumulation of protein aggregates, partly associated with disruption of synaptic function and, in some cases, with neuronal death [63]. Neuronal cells are particularly sensitive to protein misfolding and ER dysfunction has been implicated in many neurodegenerative diseases [64]. The tyrosine residue UPF3B^Y160^ is highly conserved in vertebrates, *Drosophila melanogaster*, and *Caenorhabditis elegans*. The UPF3B^Y160D^ mutation is genetically linked to XLID. UPF3B^Y160D^ significantly inhibits both the interactions of UPF3B with IRE1α and UPF2, suggesting that the missense mutation of UPF3B loses its suppressive function in IRE1α activation, potentially leading to chronic UPR activation for the development of some neurodegenerative diseases. However, the relevance of this effect to the mental retardation caused by this genetic mutation remains to be investigated in animal models. Phosphorylation of UPF3B appeared to be up-regulated under ER stress conditions, and the phosphorylation of Thr169 of UPF3B attenuated both the UPF3B-IRE1α interaction and the UPF2-UPF3B interaction. In contrast to UPF3B^WT^, UPF3B^Y160F^, and UPF3B^T169A^, restoration of various UPF3B mutants including UPF3B^Y160D^ and UPF3B^T169D^ in shUPF3B cells, failed to suppress cell apoptosis either under both normal and stress conditions, further supporting the important role of these two interactions in ER stress-related pathogenesis.

Activation of the UPR signaling pathway reduces overall protein synthesis, increases ER protein folding capacity, and promotes degradation of misfolded proteins [65]. If the UPR fails to achieve ER homeostasis in a timely manner, programmed cell death would be triggered as a cellular response. UPF3B^T169D^ and UPF3B^Y160D^, inhibit its interaction with IRE1α and UPF2, and cause the apparent cell apoptosis, providing a potential target for therapeutics to ameliorate the deleterious outcome of ER stress. For the first time, our study provides the evidence for the interplay role of UPF3B in NMD and ER stress and a new perspective on the physiological significance of NMD in modulating ER stress.

## Materials and methods

### Cell culture and transfection

HEK293T (ATCC, CRL-3216) and U2OS (ATCC, HTB-96) cell lines were preserved in our laboratory. All the cell lines were regularly tested and ensured to be negative for mycoplasma contamination. The cells were cultured in Dulbecco’s modified Eagle’s medium (DMEM, Thermo Fisher, USA) containing 10% fetal calf serum (BI, German) at 37 °C in a CO_2_ incubator (Thermo Fisher, USA), 0.25% trypsin-EDTA, and puromycin that were obtained from Invitrogen (Carlsbad, CA, USA). Lipofectamine 2000 and 3000 were obtained from Invitrogen (Carlsbad, CA, USA). According to the Lipofectamine 3000 operating instructions, plasmids, P3000 and Lipofectamine 3000 were added into the serum-free Opti-MEM and left for 5 min. Stand for 15 min after mixing, cells were added to transfection medium and cultured at 37°C for 5 h and then replaced the complete medium.

### Chemical reagents

Thapsigargin (Tg) and tunicamycin (Tm) were purchased from Beyotime Biotech (Shanghai, China). NMDI14, harringtonine, puromycin, cycloheximide, STF-083010 and Kira6 were purchased from Selleck (Shanghai, China). Hoechst 33342 and 4’,6-diamidino-2-phenylindole (DAPI) were obtained from Beyotime Biotech (Shanghai, China).

### Antibodies

The antibodies applied in this study are listed in Supplementary Table 2.

### Plasmids

The human IRE1α plasmid was purchased from Addgene (#13009), and the human UPF3B plasmid was purchased from Sino Biological (HG16941-CF). The expression plasmids for the deletion mutants and point mutations of IRE1α and UPF3B were cloned into pcDNA4 with the Flag tag in-frame at the N terminus. Flag, Myc, and HA epitope tags were added to the C-terminal coding ends of the IRE1α and UPF3B constructs. For BiFC analysis, VN173 and VC155, which are complementary fragments of Venus, were fused to the C-terminal of IRE1α and the N-terminal of UPF3B, respectively. All constructs were verified by DNA sequencing.

### Cytotoxicity assay

The cytotoxic effects of drugs were determined in indicated cell lines using a cell counting kit-8 (CCK-8) assay (Sangon Biotech, China). CCK-8 contains WST-8, which is reduced by the electron carrier (1-Methoxy PMS) to the highly water-soluble yellow formazan dye by dehydrogenase in the mitochondria. The amount of formazan produced is proportional to the number of living cells, so this property can be used for direct cell toxicity analysis. Cells were seeded in a 96-well plate at 5000 cells per well in the presence of the indicated concentration of drugs in a cell culture incubator. CCK-8 solution (0.5 mg/mL) was added to each well for 1 h at 37 °C. The optical density of each well was determined at a wavelength of 450 nm using a microplate reader (Promega, USA).

### Quantitative reverse-transcription polymerase chain reaction (qRT-PCR)

We grew the cells on a six-well plate, then isolated the RNA using TRIZOL reagents (Invitrogen, USA) according to the protocol. The cDNA was synthesized using a PrimeScript™ RT Reagent Kit according to the manufacturer’s protocol (Takara Biotechnology, China). The qRT-PCR samples were prepared using SYBR Green PCR Master Mix (Promega, USA) and the primers were listed in Supplementary Table 1. The samples were optimized for amplification under the following reaction conditions: denaturization at 95 °C for 10 min; followed by 36 cycles at 95 °C for 15 s, and 60 °C for 1 min. The melting curve of each sample was analyzed after completion of the amplification protocol. We used the housekeeping gene GAPDH for the expression control.

### Western blot

The total cell lysates were prepared by using cold radioimmunoprecipitation assay (RIPA) lysis buffer (50 mM Tris-HCl, 150 mM NaCl, 1% Triton X-100, pH 7.8) containing protease and phosphatase inhibitors on ice for 30 min, and then centrifuged the mixture for 10 min at 14 000×g. The lysates were subjected to sodium dodecyl sulfate–polyacrylamide gel electrophoresis (SDS-PAGE) and transferred to polyvinylidene fluoride membranes (Millipore, USA). The enhanced chemiluminescence mix (ECL, Beyotime Biotech, China) were used to visualize the proteins.

### shRNA-mediated gene knockdown

RNA interference was carried out by using a shRNA expressing H1 retroviral system. The RNA-mediated interference of UPF1, UPF2 and UPF3B was performed in HEK293T cells using pLKO.1 vector encoding the shRNA sequence. UPF1: 5’- CCTGCGTGGTTTACTGTAATA -3’, UPF2: 5’- CATCAGAGTCAGTGCTATAAA -3’, UPF3A: 5’- GCATCGAAGATGATCCAGAAT -3’, UPF3B: 5’- GAAGCCTTGTTCCGATCTAAT -3’, BiP: 5’- GCTCGACTCGAATTCCAAAGA -3’, respectively. Negative control was done by same way with the vector pLKO.1 containing non-hairpin insert. The knockdown efficiency of the target genes was validated by western blotting.

### Transfection of small inhibitory RNA

Cells were cultured to 70-80% confluence in 10% FBS supplemented DMEM and transfected with siRNA using Lipofectamine 3000 reagent according to the manual instruction. A non-targeting 20-25 nucleotide sequence siRNA was used as a negative control. The list of primers used for siRNA is as follows: siNC-sense: 5’-UUCUCCGAACGUGUCACGUTT -3’, siBiP-sense: 5’- CCUUCGAUGUGUCUCUUCUTT -3’, siUPF3A-sense: 5’- CCCUAGAAGUGCAGUUCCATT -3’.

### Flow cytometry assay

The cell apoptosis was detected with an Annexin-V-FITC/PI detection kit purchased from Beyotime Biotechnology (Shanghai, China). Cells were precipitated by centrifugation at 1 000 g for 5 min, and discard the supernatant. The collected cells were washed twice with cool PBS, precipitated by centrifugation and gently resuspended by adding 500 μL Annexin V buffer. 5 μL Annexin V-FITC and 5 μL propidium iodide staining solution were added and gently mixed. Incubate for 15 min at room temperature (20-25 °C) in the dark. Then the cells were immediately detected with the flow cytometer (FACSCalibur, BD, USA).

### Immunofluorescence

U2OS and HEK293T cells were cultured on cell slides inside a 24-well plate for 24 h. The medium was then decanted, and the wells were washed three times with cold PBS. The cells were then fixed in 4% paraformaldehyde for 15 min and permeabilized in 0.5% Triton X-100 for 5 min. After washing three times with PBS, the cells were blocked for 1 h in PBS with 5% bovine serum albumin. The primary antibodies were diluted by 1:100 in PBS with 1% bovine serum albumin and incubated 1 h. After washing three times with PBS, Alexa Fluor 488 anti-rabbit and Alexa Fluor 594 anti-mouse antibodies (Cell Signal Technology, USA) were added to the antibody dilution buffer at 1:500 dilutions. We then added DAPI to the slides and incubated for 2 min at room temperature. After washing the slides three times with PBS, we mounted them using an antifade reagent (Invitrogen, USA). We acquired images using a two-photon super-resolution point scanning confocal microscope (AX, Nikon, Japan) and selected representative images for each sample.

### Co-immunoprecipitation

U2OS and HEK293T cells were seeded in 60 mm culture dishes and transfected using Lipofectamine 2000 reagent (Invitrogen, USA). After transfection for 24 h, we lysed the cells in NETN buffer (20 mM Tris-HCl pH 8.0, 100 mM NaCl, 1 mM EDTA, 0.5% NP-40) with 1% protease and phosphatase inhibitor cocktail (Sigma Aldrich, USA). The cell extract was used to immunoprecipitated Flag with anti-Flag (M2) magnetic beads as described, and the beads were then washed three times with NETN buffer. We analyzed the samples by western blotting with antibodies.

### GST pulldown assay

GST, GST-UPF3B and GST-IRE1α^K^ fusion proteins were expressed in Rosetta bacterial cells using standard procedures, and subsequently incubated overnight with Glutathione Sepharose 4 s (GE Healthcare) at 4 °C while agitating the mixture. The beads were then washed and resuspended in RIPA buffer. Each lysate from the HEK293T cells was first mixed with agarose beads conjugated with GST fusion protein, then incubated for 4 h at 4 °C while rotating gently. The beads were washed three times with RIPA buffer, and the eluted protein samples were further subjected to western blotting analysis of the indicated proteins.

### BiFC analysis

We grew U2OS cells on coverslips inside a 24-well plate at 37 °C in a cell culture incubator. The UPF3B-Vc155 and IRE1α-Vn173 constructs and the mutants were transfected with Lipofectamine 2000 according to the manufacturer’s instructions. After transfection for 24 h, the nuclear DNA of the living cells was stained with Hoechst 33342. We acquired images using a two-photon super-resolution point scanning confocal microscope (AX, Nikon, Japan) and selected representative images for each sample. 40-50 cells from three independent biological experiments were randomly selected for statistical analysis.

### Statistical analysis and reproducibility

Quantitative data are expressed as the mean ± standard error of the mean (SEM) of at least three independent experiments. Statistical differences between multiple comparisons were analyzed by one-way or two-way analysis of variance (ANOVA) with Bonferroni correction where appropriate. A two-tailed unpaired t-test was used to compare the means of the two groups. All western blots and fluorescence tests were performed on at three independent biological experiments to ensure reproducibility, and representative images were shown.

## Supplementary information

Supplemental figures and tables

Original data

## Data Availability

All data supporting this study are presented in this published article and in its Supplementary information files.
