# Correction: UPF3B modulates endoplasmic reticulum stress through interaction with inositol-requiring enzyme-1α

**DOI:** 10.1038/s41419-024-07033-6

**Published:** 2024-09-18

**Authors:** Xingsheng Sun, Ruqin Lin, Xinxia Lu, Zhikai Wu, Xueying Qi, Tianqing Jiang, Jun Jiang, Peiqiang Mu, Qingmei Chen, Jikai Wen, Yiqun Deng

**Affiliations:** 1 State Key Laboratory of Swine and Poultry Breeding Industry, South China Agricultural University, Guangzhou, 510642 Guangdong China; 2 Guangdong Academy of Agricultural Sciences, Guangzhou, 510640 Guangdong China; 3 Guangdong provincial key laboratory for the development biology and environmental adaptation of agricultural organisms, South China Agricultural University, Guangzhou, 510642 Guangdong China

**Keywords:** Endoplasmic reticulum, Stress signalling

*Correction to: Cell Death and Disease* 10.1038/s41419-024-06973-3, published online 13 August 2024

In this article, the authors have been affiliated erroneously:

XingSheng Sun^1,2^, Ruqin Lin^1,2^, Xinxia Lu^1,2^, Zhikai Wu^1,2^, Xueying Qi^1,2^, Tianqing Jiang^1,2^, Jun Jiang^1,2^, Peiqiang Mu^1,2^, Qingmei Chen^1,2^, Jikai Wen^1,2,*^ and Yiqun Deng^1,3^

^1^State Key Laboratory of Swine and Poultry Breeding Industry, South China Agricultural University, Guangzhou 510642 Guangdong, China.

^2^Guangdong provincial key laboratory for the development biology and environmental adaptation of agricultural organisms, South China Agricultural University, Guangzhou 510642 Guangdong, China.

^3^Guangdong Academy of Agricultural Sciences, Guangzhou 510640 Guangdong, China

It should read:

Xingsheng Sun^1,3^, Ruqin Lin^1,3^, Xinxia Lu^1,3^, Zhikai Wu^1,3^, Xueying Qi^1,3^, Tianqing Jiang^1,3^, Jun Jiang^1,3^, Peiqiang Mu^1,3^, Qingmei Chen^1,3^, Jikai Wen^1,3^ and Yiqun Deng^1,2^

^1^State Key Laboratory of Swine and Poultry Breeding Industry, South China Agricultural University, Guangzhou 510642 Guangdong, China.

^2^Guangdong Academy of Agricultural Sciences, Guangzhou 510640 Guangdong, China.

^3^Guangdong provincial key laboratory for the development biology and environmental adaptation of agricultural organisms, South China Agricultural University, Guangzhou 510642 Guangdong, China.

The original article has been corrected.

