# Supplementary material for: UPF3B modulates endoplasmic reticulum stress through interaction with inositol-requiring enzyme-1α
Source: Cell Death Dis. 2024 Aug 13;15(8):587. doi: 10.1038/s41419-024-06973-3 (PMC11322666; doi:10.1038/s41419-024-06973-3)
Supplement: Supplementary file 2 — Original data [file 41419_2024_6973_MOESM2_ESM.docx]

| **Fig. 1A NMDI14** |  |  |  |
| --- | --- | --- | --- |
| **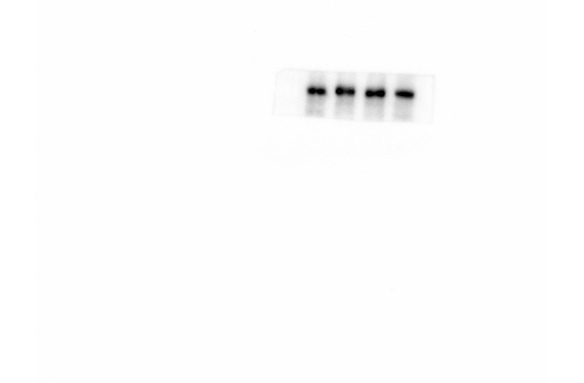** | **PERK** | **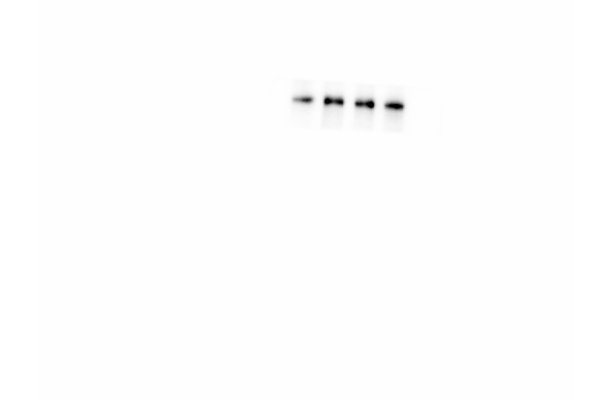** | **p-PERK** |
| **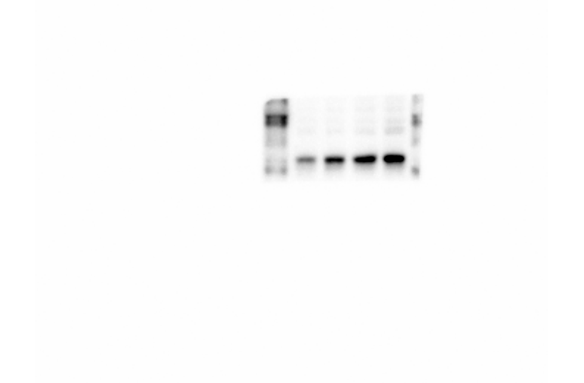** | **p-eIF2α** | **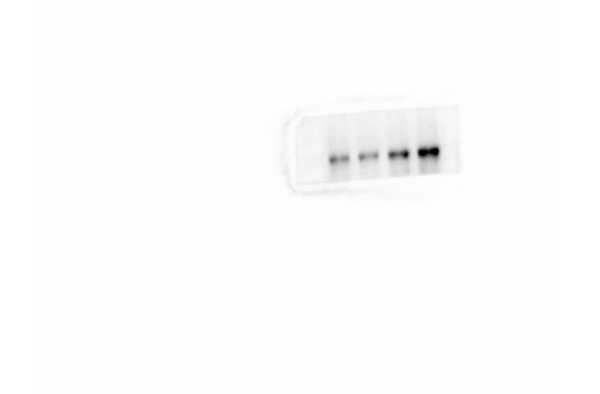** | **IRE1α** |
| **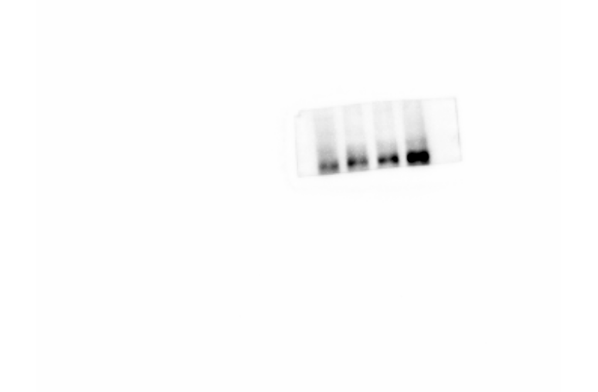** | **p-IRE1α** | **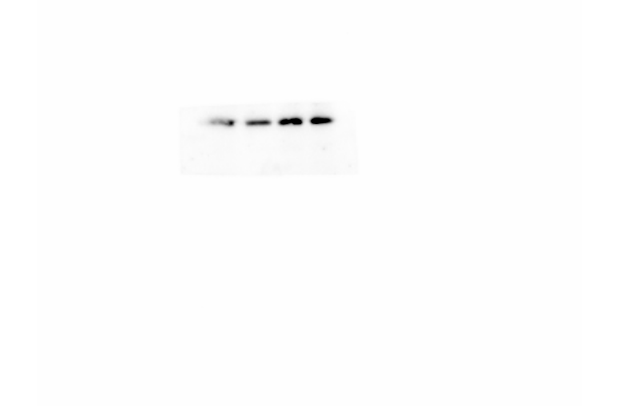** | **XBP1s** |
| **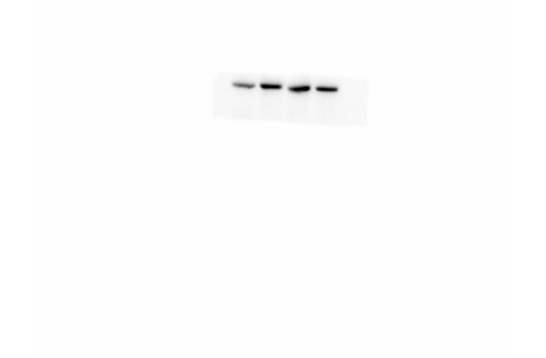** | **ATF6** | **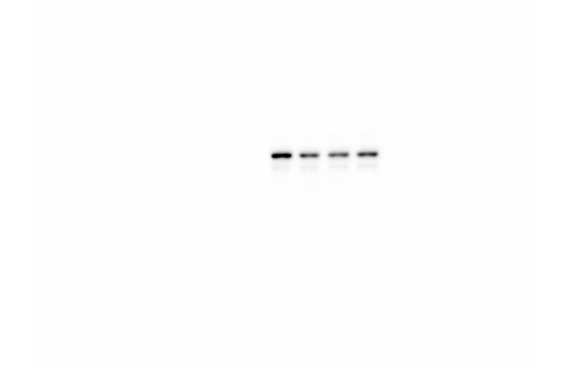** | **BiP** |
| **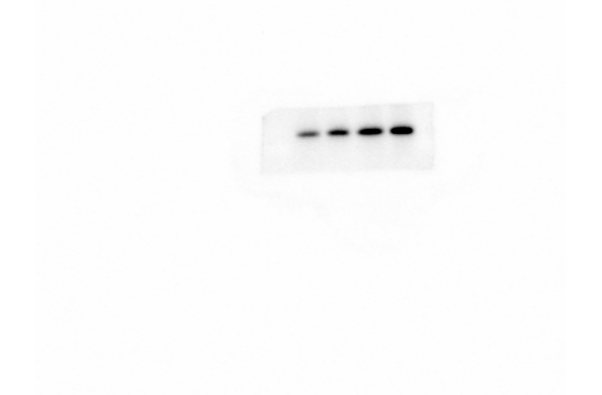** | **CHOP** | **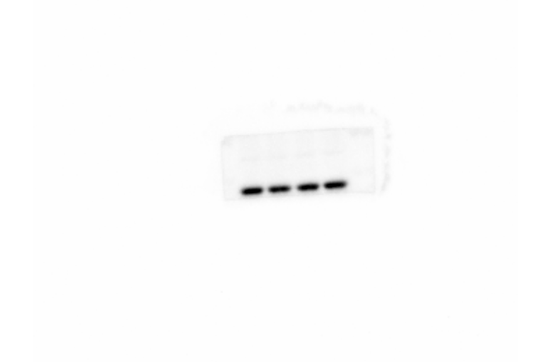** | **GAPDH** |
| **Fig.1A HHT** |  |  |  |
| **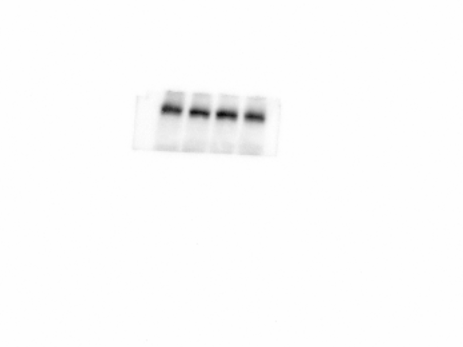** | **PERK** | **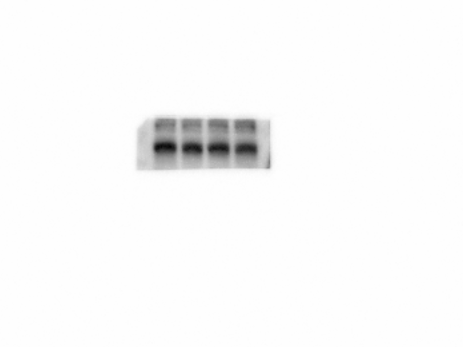** | **p-PERK** |
| **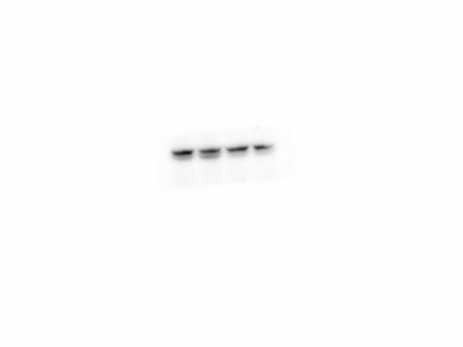** | **p-eIF2α** | **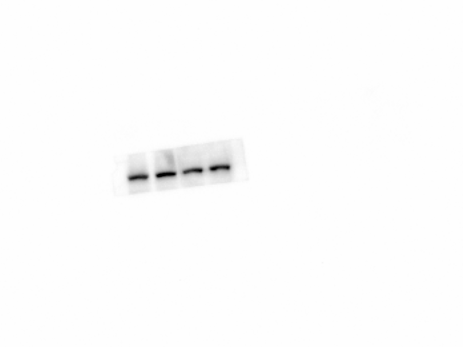** | **IRE1α** |
| **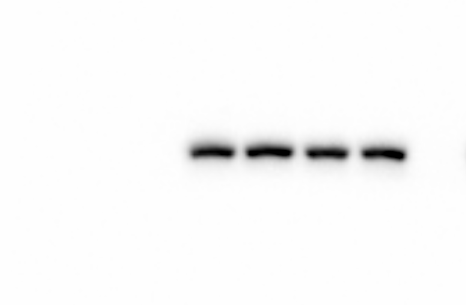** | **p-IRE1α** | **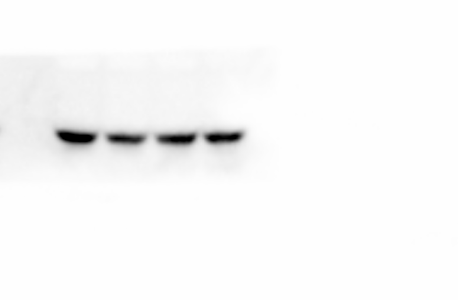** | **XBP1s** |
| **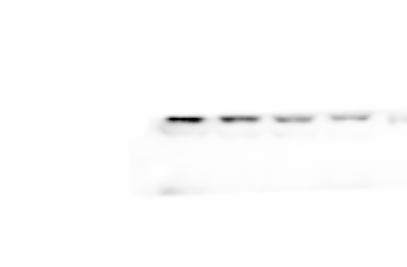** | **ATF6** | **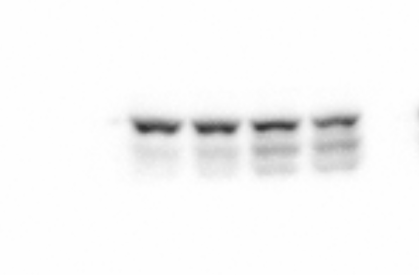** | **BiP** |
| **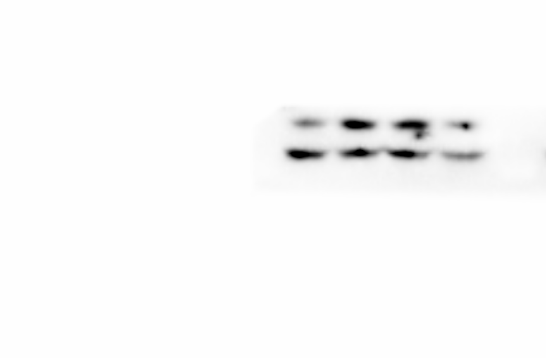** | **CHOP** | **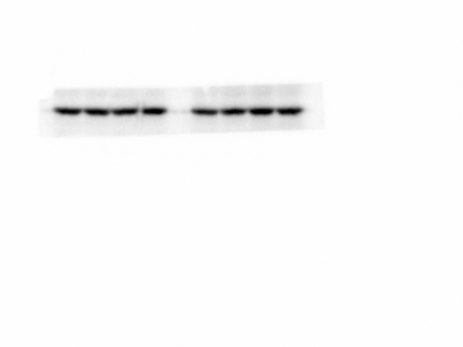** | **GAPDH** |
| **Fig. 1A Puro** |  |  |  |
| **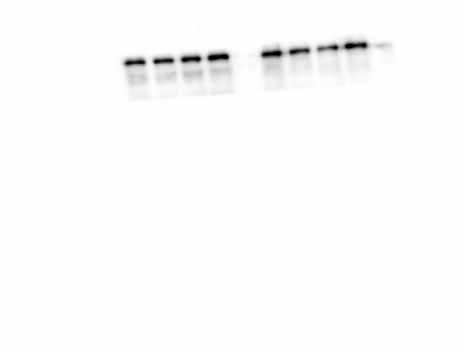** | **PERK** | **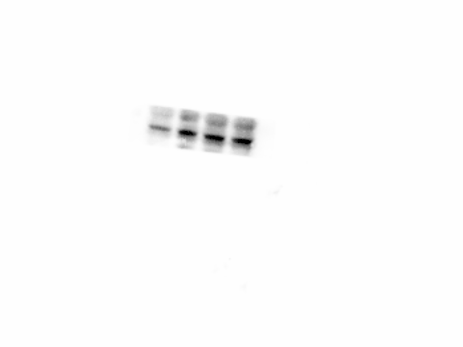** | **p-PERK** |
| **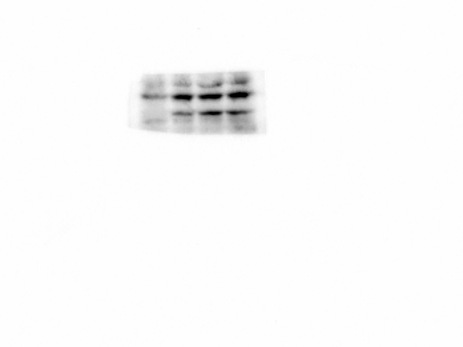** | **p-eIF2α** | **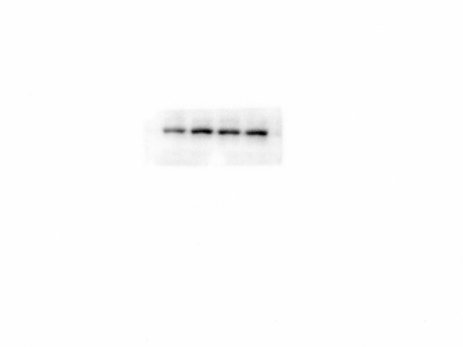** | **IRE1α** |
| **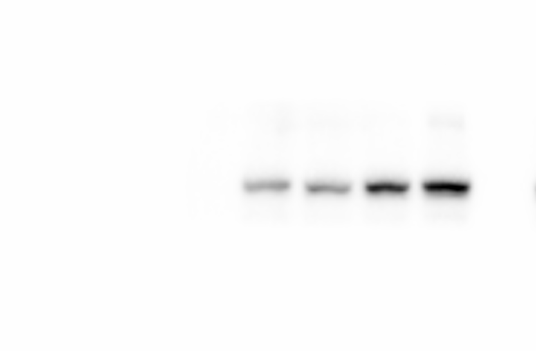** | **p-IRE1α** | **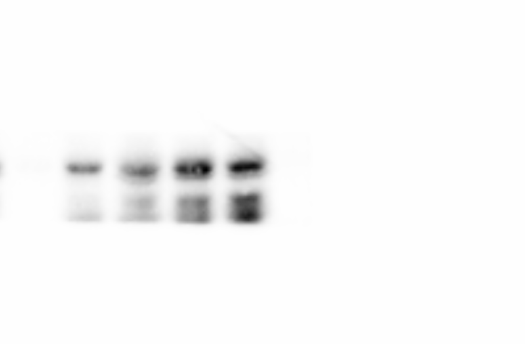** | **XBP1s** |
| **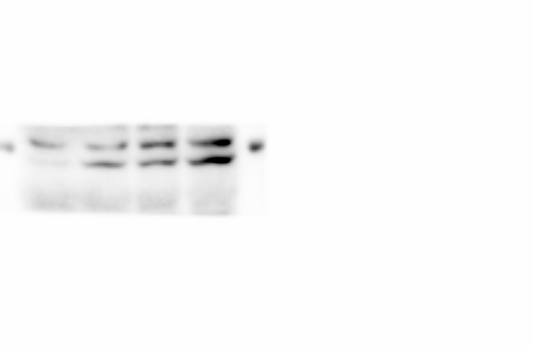** | **ATF6** | **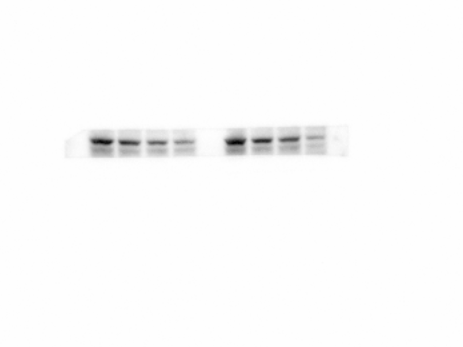** | **BiP** |
| **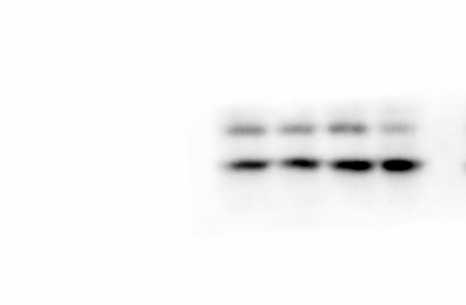** | **CHOP** | **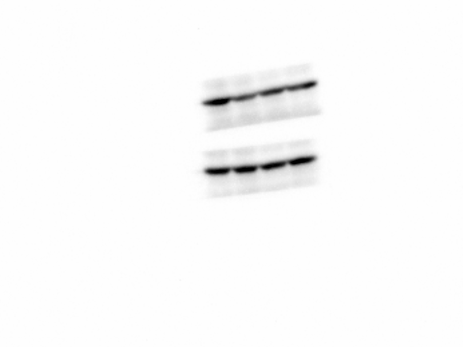** | **GAPDH** |
| **Fig. 1A CHX** |  |  |  |
| **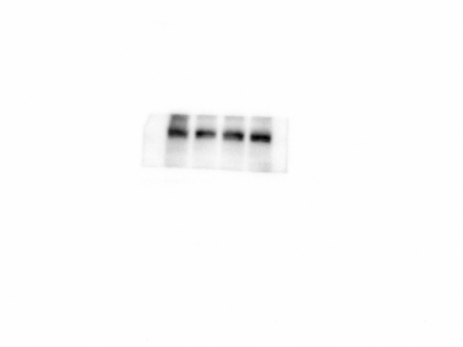** | **PERK** | **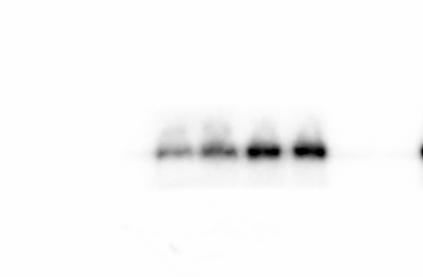** | **p-PERK** |
| **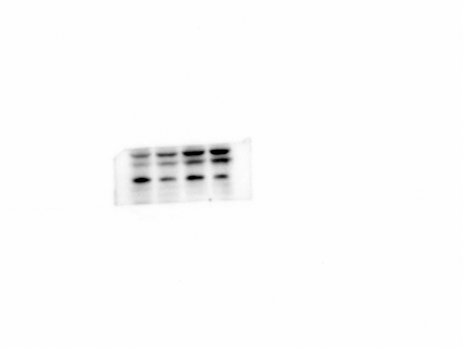** | **p-eIF2α** | **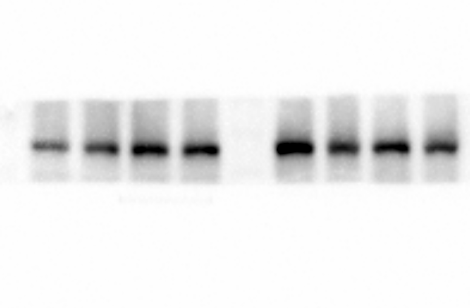** | **IRE1α** |
| **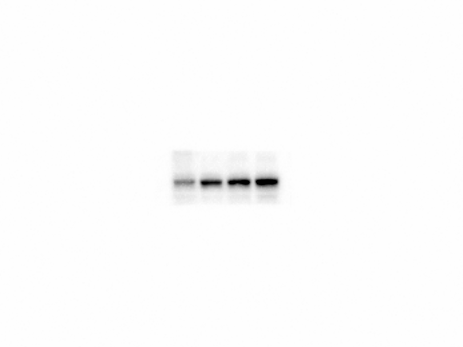** | **p-IRE1α** | **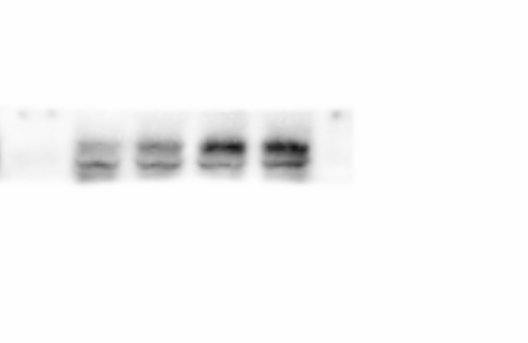** | **XBP1s** |
| **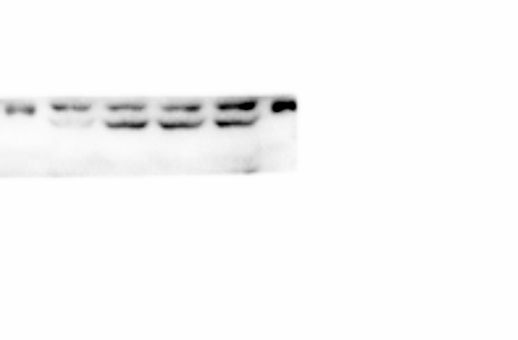** | **ATF6** | **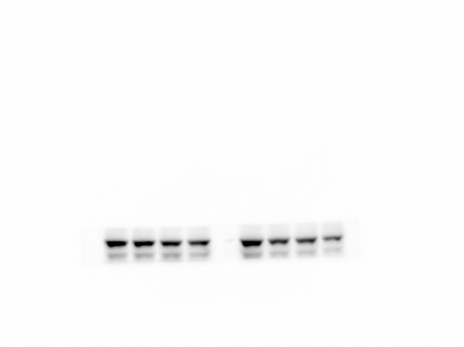** | **BiP** |
| **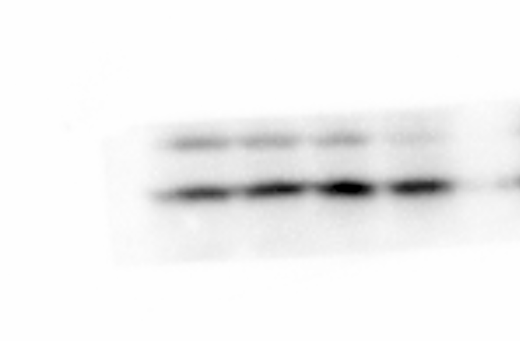** | **CHOP** | **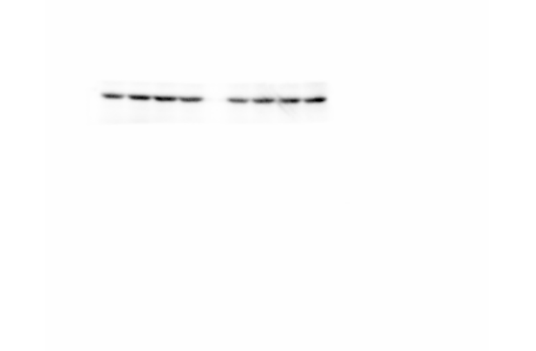** | **GAPDH** |
| **Fig. 1F** |  |  |  |
| **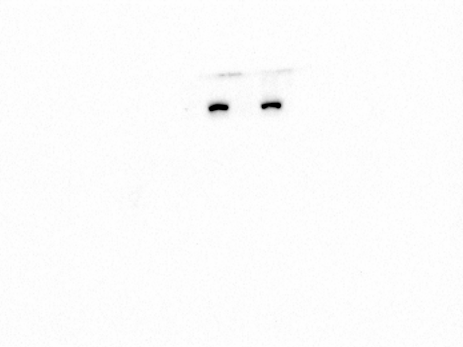** | **UPF1** | **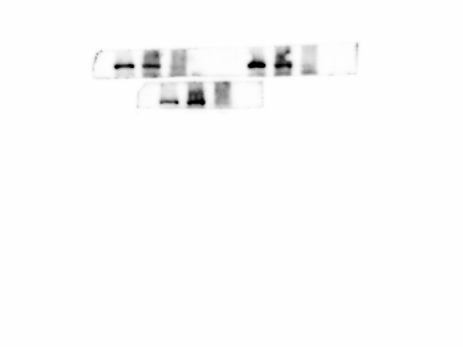** | **UPF2** |
| **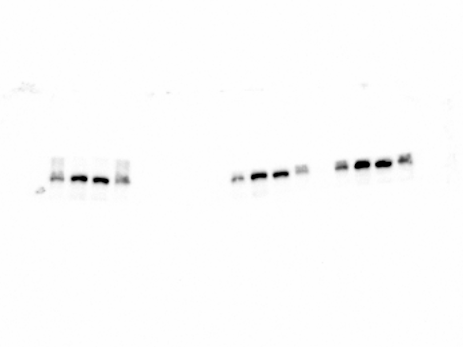** | **PERK** | **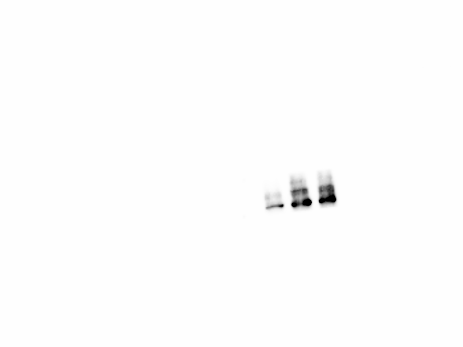** | **p-PERK** |
| **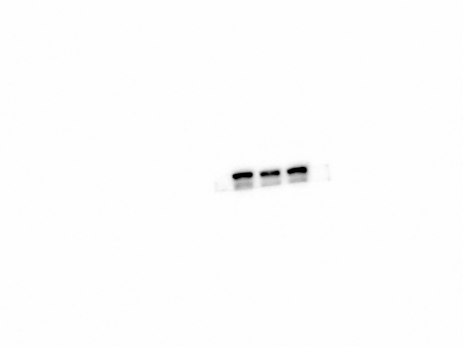** | **IRE1α** | **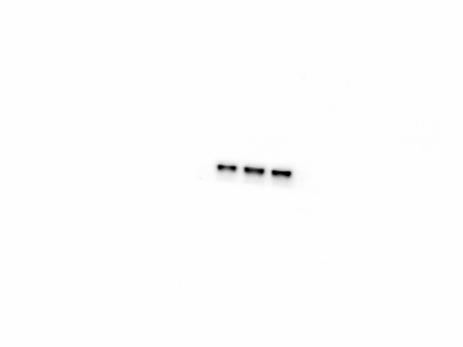** | **p-IRE1α** |
| **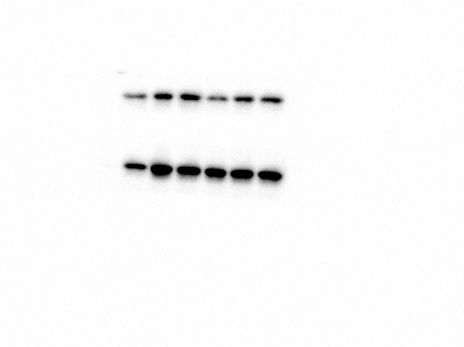** | **p-eIF2α** | **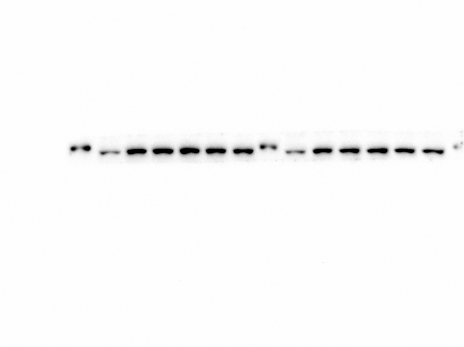** | **ATF6** |
| **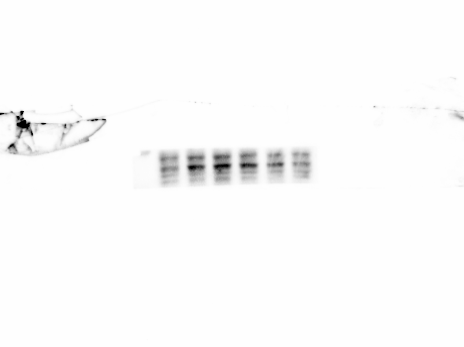** | **XBP1s** | **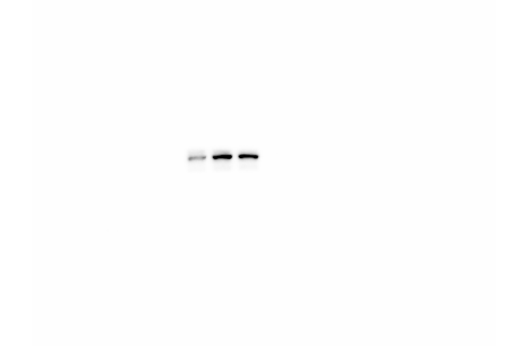** | **BiP** |
| **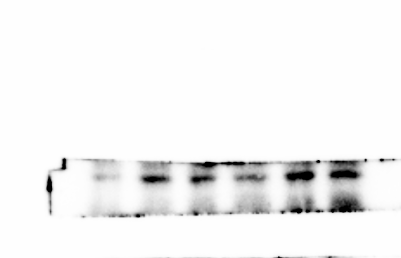** | **CHOP** | **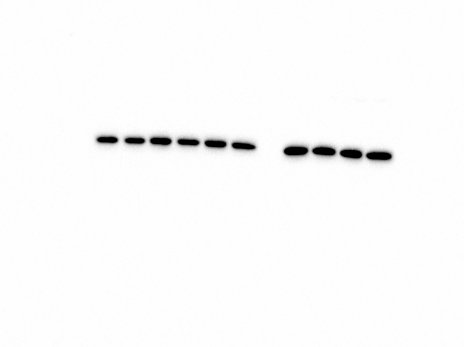** | **GAPDH** |
| **Fig. 1G** |  |  |  |
| **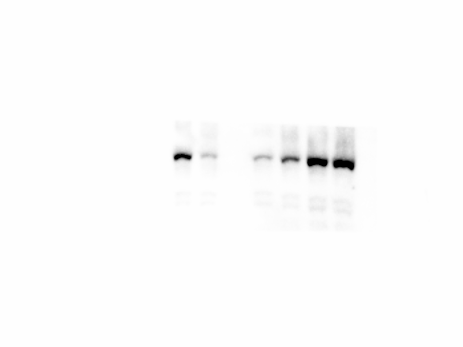** | **UPF3B** | **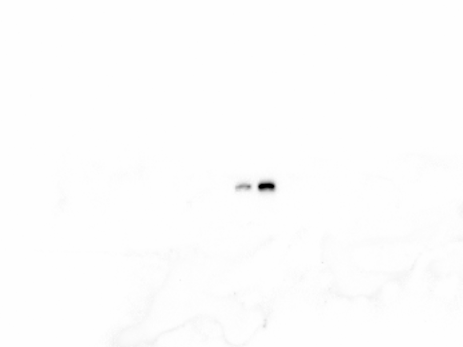** | **PERK** |
| **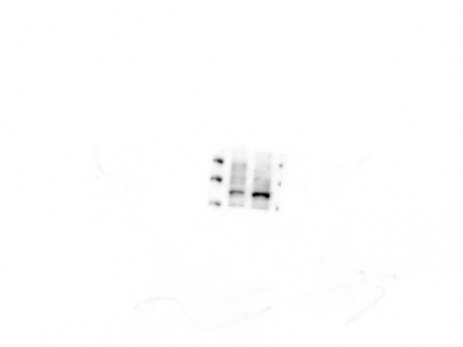** | **p-PERK** | **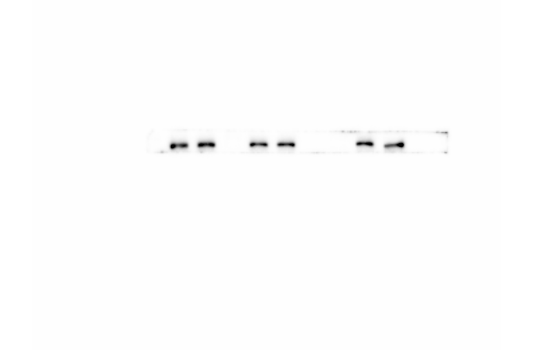** | **IRE1α** |
| **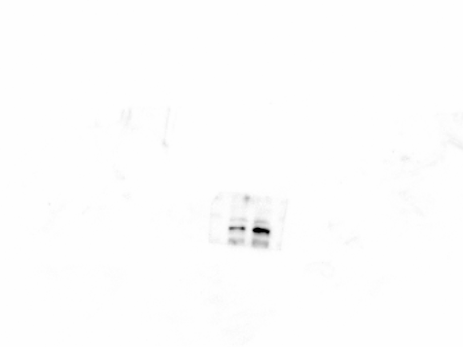** | **p-IRE1α** | **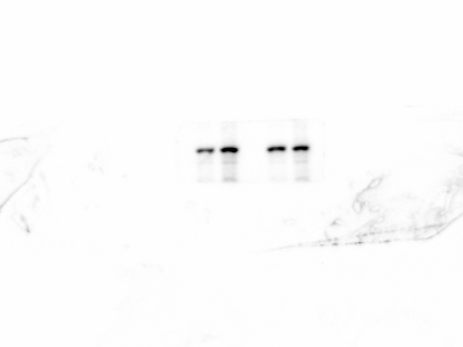** | **p-eIF2α** |
| **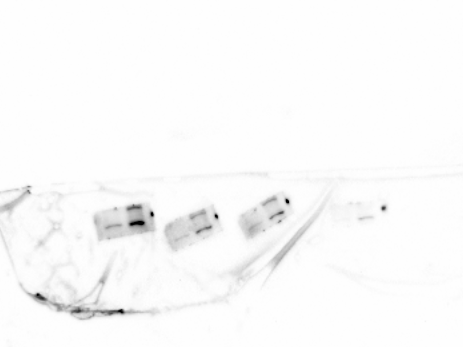** | **ATF6** | **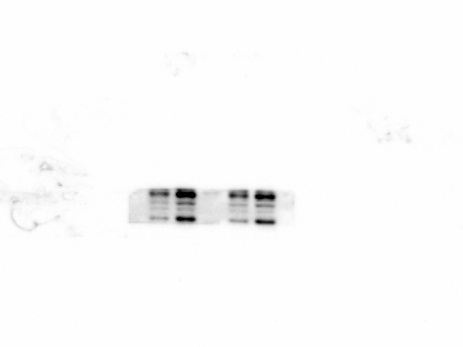** | **XBP1s** |
| **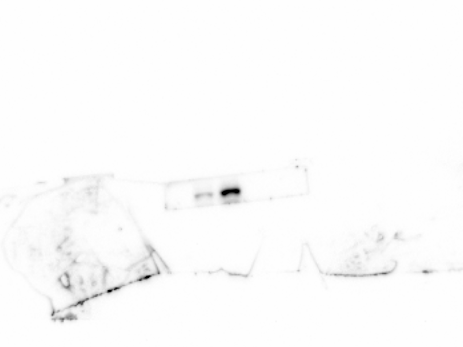** | **BiP** | **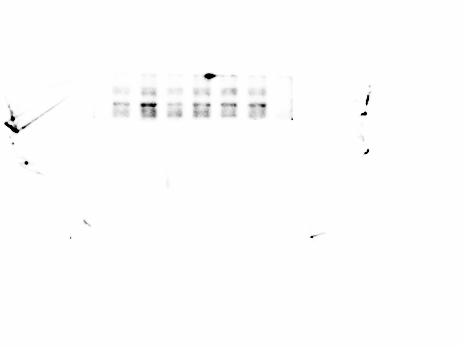** | **CHOP** |
| **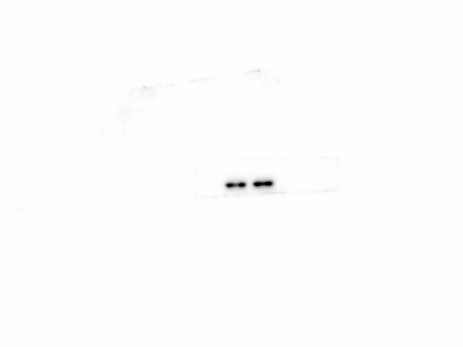** | **GAPDH** |  |  |
| **Fig. 2A** |  |  |  |
| **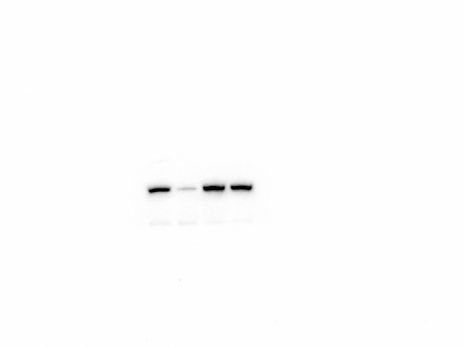** | **UPF1** | **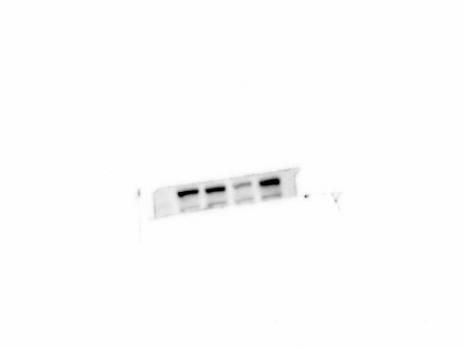** | **UPF2** |
| **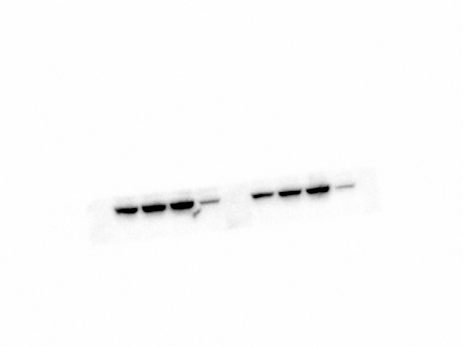** | **UPF3B** | **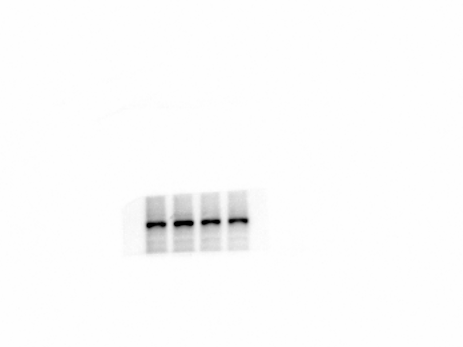** | **IRE1α** |
| **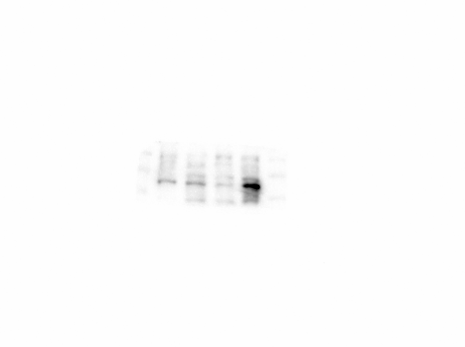** | **p-IRE1α** | **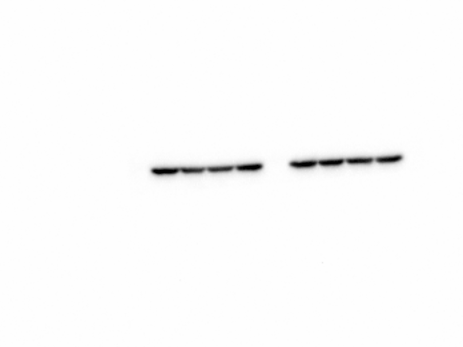** | **GAPDH** |
| **Fig. 2B** |  |  |  |
| **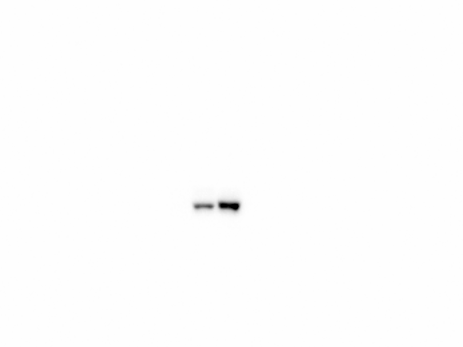** | **UPF3B** | **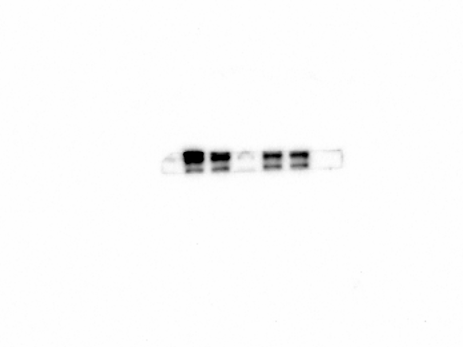** | **IRE1α** |
| **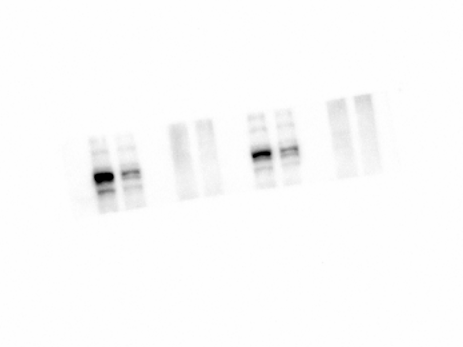** | **p-IRE1α** | **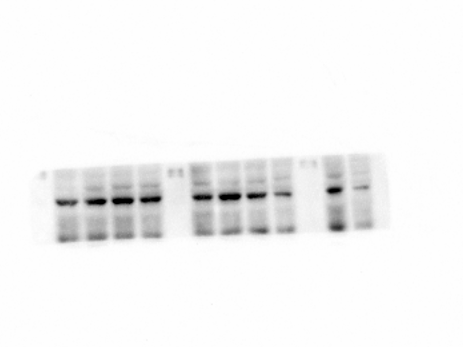** | **XBP1s** |
| **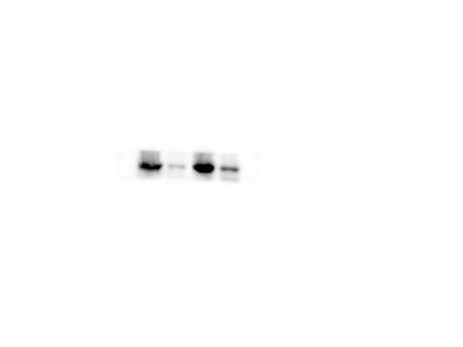** | **BiP** | **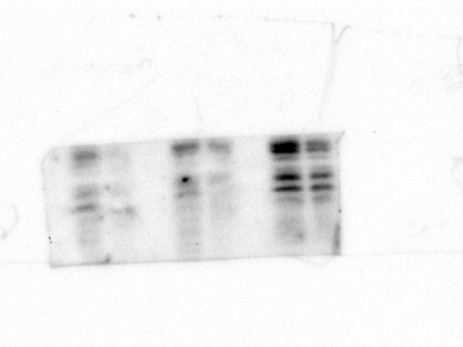** | **CHOP** |
| **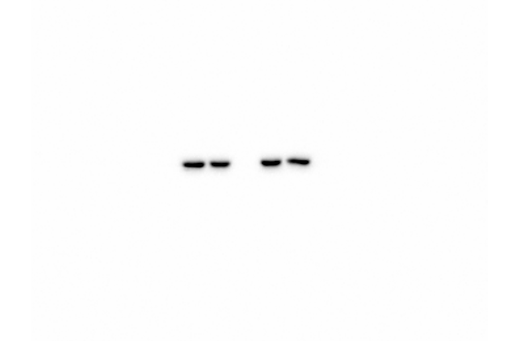** | **GAPDH** | **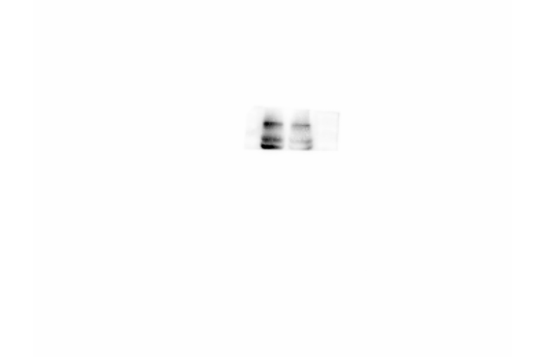** | **p-PERK** |
| **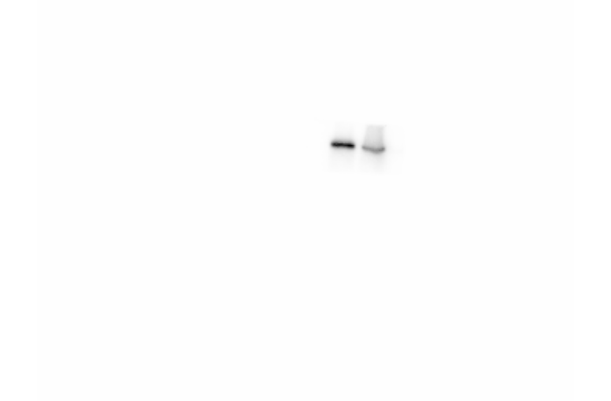** | **PERK** | **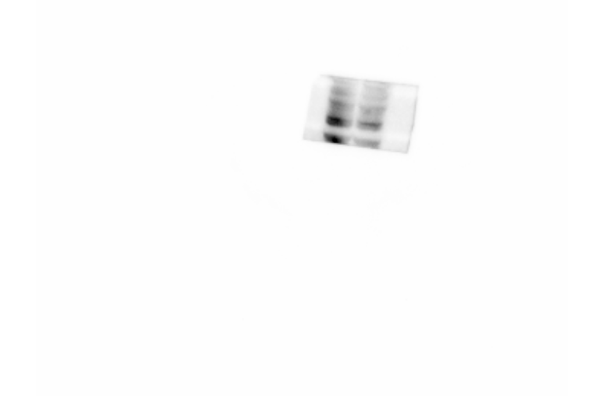** | **ATF4** |
| **Fig. 2F IP: IRE1α** |  |  |  |
| **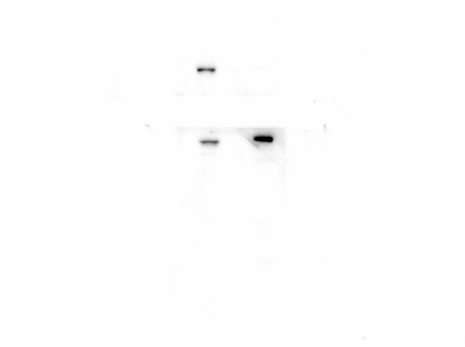** | **IRE1α** | **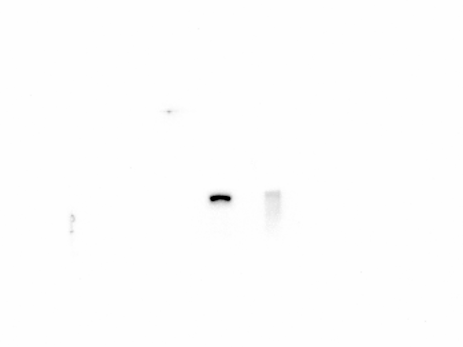** | **UPF1** |
| **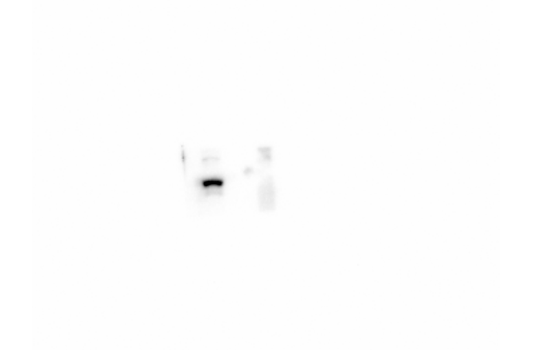** | **UPF2** | **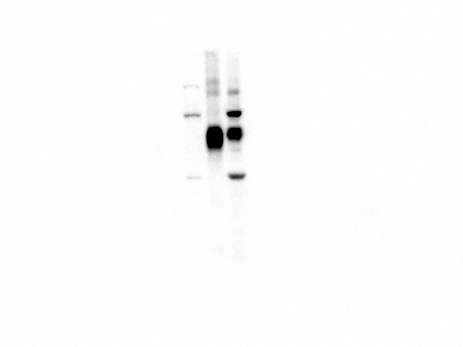** | **UPF3B** |
| **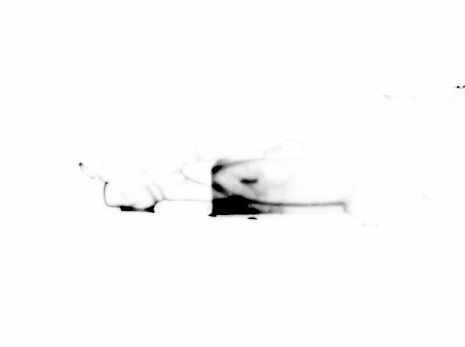** | **GAPDH** |  |  |
| **Fig. 2F IP: UPF3B** |  |  |  |
| **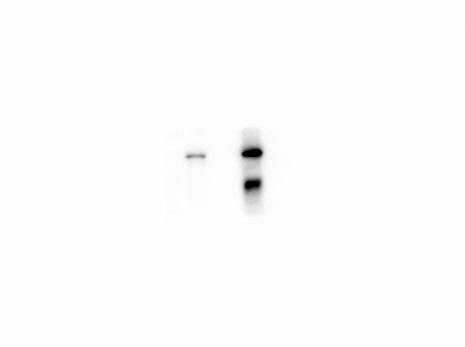** | **UPF3B** | **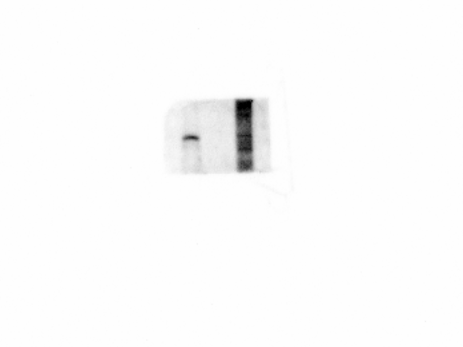** | **IRE1α** |
| **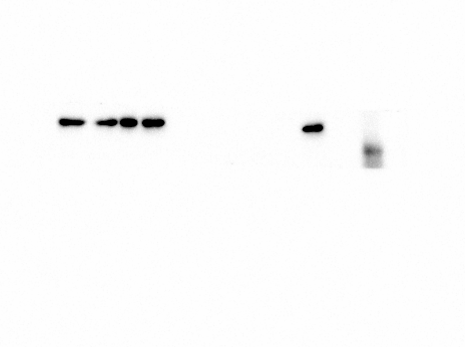** | **GAPDH** |  |  |
| **Fig. 2G IP: IRE1α** |  |  |  |
| **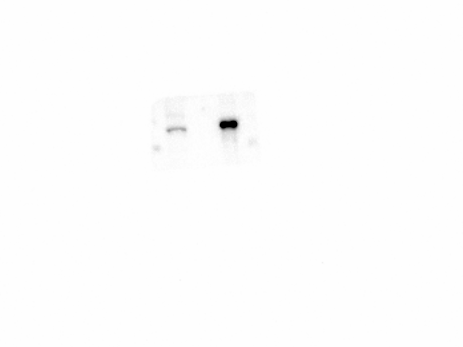** | **IRE1α** | **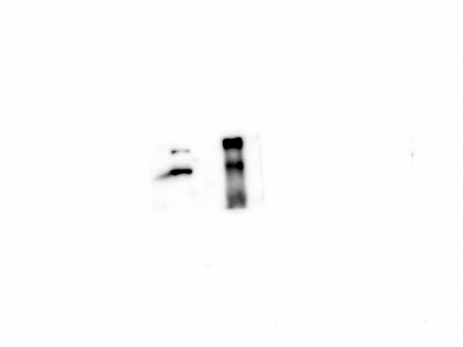** | **UPF3B** |
| **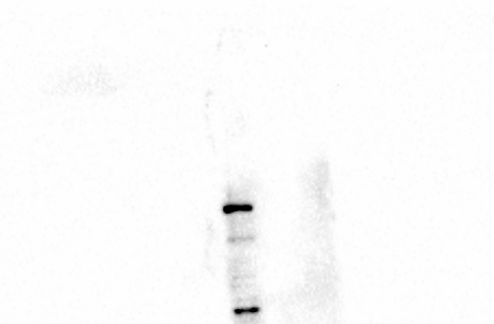** | **GAPDH** |  |  |
| **Fig. 2F IP: UPF3B** |  |  |  |
| **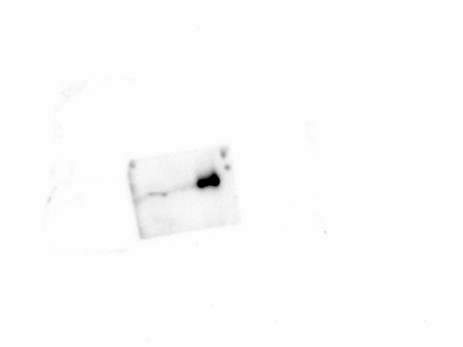** | **UPF3B** | **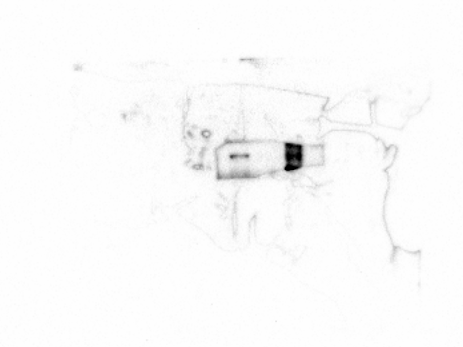** | **IRE1α** |
| **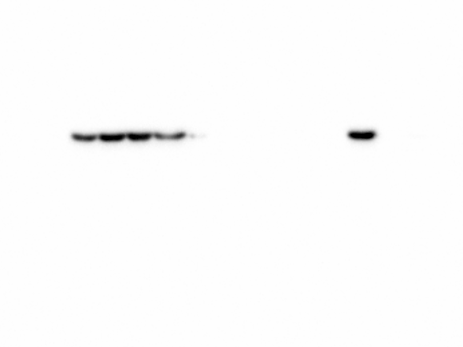** | **GAPDH** |  |  |
| **Fig. 3C** |  |  |  |
| **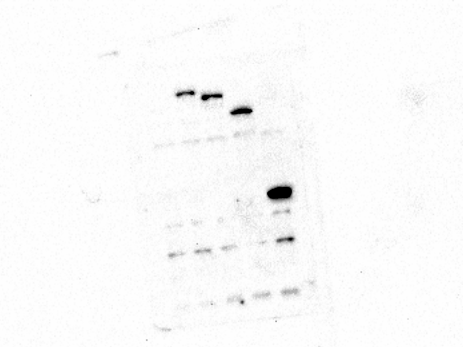** | **1% Input**  **Flag(IRE1α)** | **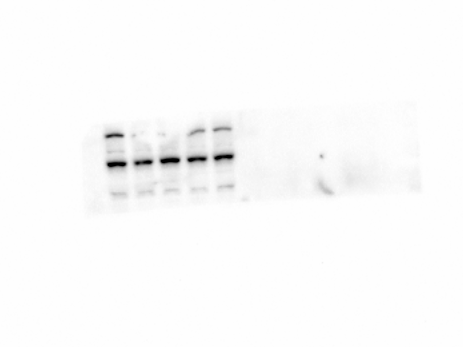** | **1% Input**  **UPF3B** |
| **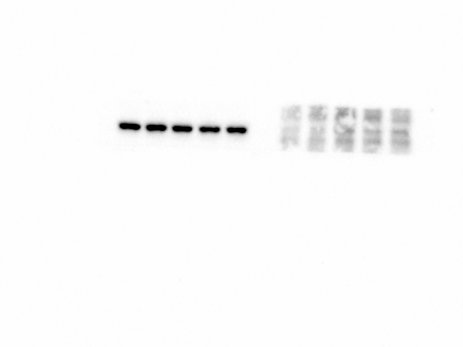** | **1% Input**  **GAPDH** | **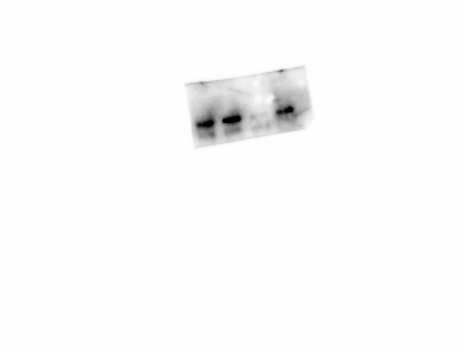** | **IP: Flag**  **UPF3B** |
| **Fig. 3D** |  |  |  |
| **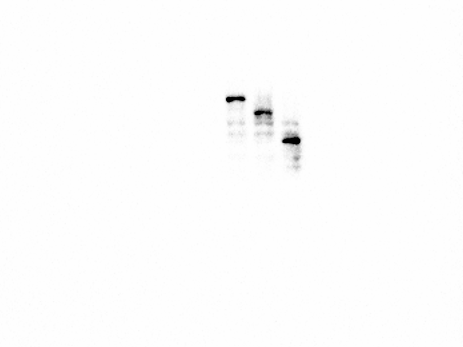** | **Input: Flag 1** | **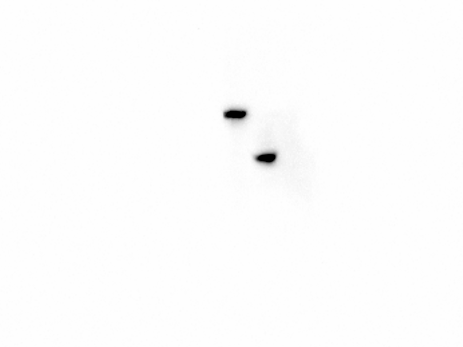** | **Input: Flag 2** |
| **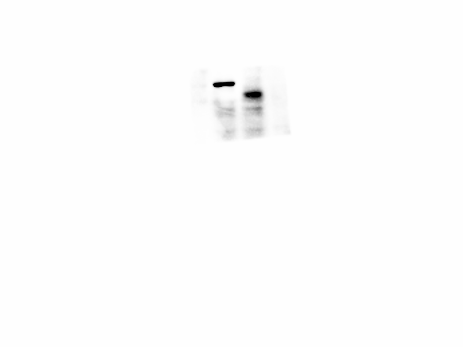** | **Pulldown: Flag 1** |  | **Pulldown: Flag 2** |
|  | **GST** |  |  |
| **Fig. 3E** |  |  |  |
|  | **1% Input**  **UFP3B(Flag)** |  | **1% Input**  **IRE1α** |
|  | **1% Input**  **GAPDH** |  | **IP: Flag**  **IRE1α** |
| **Fig. 3F** |  |  |  |
|  | **Input: Flag** |  | **Pulldown: Flag** |
|  | **GST** |  |  |
| **Fig. 3G IP:Flag** |  |  |  |
|  | **UPF3B^RRM^(Flag)** |  | **IRE1α^k^(HA)** |
|  | **GAPDH** |  |  |
| **Fig. 3G IP:HA** |  |  |  |
|  | **IRE1α^k^(HA)** |  | **UPF3B^RRM^(Flag)** |
|  | **GAPDH** |  |  |
| **Fig. 4A** |  |  |  |
|  | **IRE1α** |  | **p-IRE1α** |
|  | **UPF3B** |  | **GAPDH** |
| **Fig. 4B** |  |  |  |
|  | **1% Input**  **IRE1α** |  | **1% Input**  **UPF3B** |
|  | **1% Input**  **p- IRE1α** |  | **1% Input**  **XBP1s** |
|  | **1% Input**  **GAPDH** |  | **IP: IRE1α**  **IRE1α** |
|  | **IP: IRE1α**  **UPF3B** |  |  |
| **Fig. 4C** |  |  |  |
|  | **1% Input**  **IRE1α** |  | **1% Input**  **UPF3B** |
|  | **1% Input**  **p- IRE1α** |  | **1% Input**  **XBP1s** |
|  | **1% Input**  **GAPDH** |  |  |
|  | **IP: IRE1α**  **UPF3B** |  | **IP: IRE1α**  **IRE1α** |
| **Fig. 4E** |  |  |  |
|  | **1% Input**  **UPF3B** |  | **1% Input**  **IRE1α** |
|  | **1% Input**  **p- IRE1α** |  | **1% Input**  **XBP1s** |
|  | **1% Input**  **GAPDH** |  | **IP: IRE1α**  **IRE1α** |
|  | **IP: IRE1α**  **UPF3B** |  |  |
| **Fig. 4F** |  |  |  |
|  | **1% Input**  **IRE1α** |  | **1% Input**  **UPF3B** |
|  | **1% Input**  **p- IRE1α** |  | **1% Input**  **XBP1s** |
|  | **1% Input**  **GAPDH** |  | **IP: Flag**  **Flag** |
|  | **IP: Flag**  **UPF3B** |  | **Input**  **IRE1α** |
| **Fig. 4G** |  |  |  |
|  | **1% Input**  **Flag(IRE1α)** |  | **1% Input**  **UPF3B** |
|  | **1% Input**  **p- IRE1α** |  | **1% Input**  **GAPDH** |
|  | **IP: Flag**  **Flag** |  | **IP: Flag**  **UPF3B** |
|  | **1% Input**  **IRE1α** |  |  |
| **Fig. 4H** |  |  |  |
|  | **1% Input**  **Flag(IRE1α)** |  | **1% Input**  **UPF3B** |
|  | **1% Input**  **p- IRE1α** |  | **1% Input**  **GAPDH** |
|  | **IP: Flag**  **Flag** |  | **IP: Flag**  **UPF3B** |
|  | **1% Input**  **IRE1α** |  |  |
| **Fig. 5B 1%Input** |  |  |  |
|  | **IRE1ɑ** |  | **p-IRE1ɑ** |
|  | **BiP** |  | **GAPDH** |
| **Fig. 5B IP** |  |  |  |
|  | **IRE1ɑ** |  | **BiP** |
| **Fig. 5D 1%Input** |  |  |  |
|  | **BiP** |  | **IRE1ɑ** |
|  | **UPF3B** |  | **p-IRE1ɑ** |
|  | **GAPDH** |  |  |
| **Fig. 5D IP** |  |  |  |
|  | **IRE1ɑ** |  | **UPF3B** |
| **Fig. 5F 1%Input** |  |  |  |
|  | **BiP** |  | **IRE1ɑ** |
|  | **UPF3B** |  | **p-IRE1ɑ** |
|  | **GAPDH** |  |  |
| **Fig. 5F IP** |  |  |  |
|  | **IRE1ɑ** |  | **UPF3B** |
| **Fig. 5G 1%Input** |  |  |  |
|  | **UPF3B** |  | **IRE1ɑ** |
|  | **p-IRE1ɑ** |  | **XBP1s** |
|  | **BiP** |  | **GAPDH** |
| **Fig. 5G IP** |  |  |  |
|  | **IRE1ɑ** |  | **BiP** |
| **Fig. 5I 1%Input** |  |  |  |
|  | **UPF3B** |  | **IRE1ɑ** |
|  | **p-IRE1ɑ** |  | **XBP1s** |
|  | **BiP** |  | **GAPDH** |
| **Fig. 5I IP** |  |  |  |
|  | **IRE1ɑ** |  | **BiP** |
| **Fig. 5L 1%Input** |  |  |  |
|  | **UPF2-Flag** |  | **IRE1ɑ** |
|  | **p-IRE1ɑ** |  | **UPF3B** |
|  | **GAPDH** |  |  |
| **Fig. 5L IP** |  |  |  |
|  | **IRE1ɑ** |  | **UPF3B** |
| **Fig. 5M 1%Input** |  |  |  |
|  | **UPF3B** |  | **UPF2** |
|  | **IRE1ɑ** |  | **p-IRE1ɑ** |
|  | **GAPDH** |  | **Flag** |
| **Fig. 5M IP** |  |  |  |
|  | **UPF3B-Flag** |  | **UPF2** |
|  | **IRE1ɑ** |  |  |
| **Fig. 6C** |  |  |  |
|  | **UPF3B** |  | **GAPDH** |
| **Fig. 6G 1%Input** |  |  |  |
|  | **Myc-UPF3B** |  | **Flag-IRE1ɑ** |
|  | **HA- IRE1ɑ** |  | **GAPDH** |
|  | **IP:Flag**  **Flag-IRE1ɑ** |  | **IP:Flag**  **HA-IRE1ɑ** |
| **Fig. 7A 1%Input** |  |  |  |
|  | **IRE1ɑ** |  | **UPF3B-Flag** |
|  | **p-IRE1ɑ** |  | **GAPDH** |
| **Fig. 7A IP: IRE1ɑ** |  |  |  |
|  | **IRE1ɑ** |  | **UPF3B** |
| **Fig.7A IP:Flag** |  |  |  |
|  | **UPF3B** |  | **IRE1ɑ** |
| **Fig. 7B 1%Input** |  |  |  |
|  | **IRE1ɑ** |  | **UPF3B-Flag** |
|  | **p-IRE1ɑ** |  | **GAPDH** |
|  | **IP:IRE1ɑ**  **IRE1ɑ** |  | **IP:IRE1ɑ**  **UPF3B** |
|  | **IP:Flag**  **IRE1ɑ** |  | **IP:Flag**  **UPF3B** |
| **Fig. 7C 1%Input** |  |  |  |
|  | **UPF3B** |  | **IRE1ɑ** |
|  | **p-IRE1ɑ** |  | **GAPDH** |
| **Fig. 7C IP** |  |  |  |
|  | **UPF3B** |  | **p-S/T** |
|  | **p-UPF3B** |  |  |
| **Fig. 7D 1%Input** |  |  |  |
|  | **UPF3B** |  | **IRE1ɑ** |
|  | **p-IRE1ɑ** |  | **GAPDH** |
| **Fig. 7D IP** |  |  |  |
|  | **UPF3B** |  | **p-S/T** |
| **Fig.7E 1%Input** |  |  |  |
|  | **UPF3B** |  | **IRE1ɑ** |
|  | **p-IRE1ɑ** |  | **GAPDH** |
| **Fig.7D IP** |  |  |  |
|  | **UPF3B** |  | **p-S/T** |
| **Fig.7G 1%Input** |  |  |  |
|  | **UPF3B-Flag** |  | **IRE1ɑ** |
|  | **p-IRE1ɑ** |  | **GAPDH** |
| **Fig. 7G IP** |  |  |  |
|  | **UPF3B-Flag** |  | **IRE1ɑ** |
| **Fig.7H 1%Input** |  |  |  |
|  | **UPF3B-Flag** |  | **IRE1ɑ** |
|  | **p-IRE1ɑ** |  | **GAPDH** |
| **Fig.7H IP** |  |  |  |
|  | **UPF3B-Flag** |  | **IRE1ɑ** |
| **Fig.7I 1%Input** |  |  |  |
|  | **UPF3B** |  | **IRE1ɑ** |
|  | **p-IRE1ɑ** |  | **GAPDH** |
| **Fig. 7I IP** |  |  |  |
|  | **UPF3B** |  | **IRE1ɑ** |
| **Fig. 7J 1%Input** |  |  |  |
|  | **UPF3B-Flag** |  | **UPF2** |
|  | **IRE1ɑ** |  | **GAPDH** |
| **Fig. 7J IP** |  |  |  |
|  | **UPF3B-Flag** |  | **UPF2** |
| **Fig. 7K 1%Input** |  |  |  |
|  | **UPF3B-Flag** |  | **UPF2** |
|  | **IRE1ɑ** |  | **GAPDH** |
| **Fig.7K IP** |  |  |  |
|  | **UPF3B-Flag** |  | **UPF2** |
| **Fig.7L 1%Input** |  |  |  |
|  | **UPF3B-Flag** |  | **UPF2** |
|  | **IRE1ɑ** |  | **GAPDH** |
| **Fig. 7L IP** |  |  |  |
|  | **UPF3B-Flag** |  | **UPF2** |

| **Figure S2** |  |  |  |
| --- | --- | --- | --- |
|  | **Input IRE1α** |  | **IP IRE1α** |
|  | **Input: p-IRE1α** |  | **IP: p-IRE1α** |
|  | **Input UPF3B** |  | **IP UPF3B** |
|  | **GAPDH** |  |  |
| **Figure S4** |  |  |  |
|  | **Input UPF1** |  | **Input UPF2** |
|  | **Input IRE1α** |  | **GAPDH** |
|  | **IP IRE1α** |  | **IP UPF3B** |
| **Figure S6A** |  |  |  |
|  | **Input IRE1α** |  | **IP IRE1α** |
|  | **GAPDH** |  | **Input HA** |
|  | **IP HA** |  |  |
| **Figure S6B** |  |  |  |
|  | **UPF3A** |  | **UPF3B** |
|  | **IRE1α** |  | **p-IRE1α** |
|  | **GAPDH** |  |  |
| **Figure S6C** |  |  |  |
|  | **UPF3A** |  | **UPF3B** |
|  | **IRE1α** |  | **p-IRE1α** |
|  | **GAPDH** |  |  |
| **Figure S7** |  |  |  |
|  | **Flag(UPF3B)** |  | **IRE1α** |
|  | **p-IRE1α** |  | **XBP1s** |
|  | **GAPDH** |  |  |
| **Figure S10A** |  |  |  |
|  | **UPF3B** |  | **IRE1α** |
|  | **BiP** |  | **p-IRE1α** |
|  | **XBP1s** |  | **GAPDH** |
| **Figure S10C** |  |  |  |
|  | **IRE1α** |  | **p-IRE1α** |
|  | **BiP** |  | **UPF3B** |
|  | **XBP1s** |  | **GAPDH** |
| **Figure S10D** |  |  |  |
|  | **IRE1α** |  | **p-IRE1α** |
|  | **BiP** |  | **UPF3B** |
|  | **XBP1s** |  | **GAPDH** |
| **Figure S11** |  |  |  |
|  | **Input Flag** |  | **Input IRE1α** |
|  | **p-IRE1α** |  | **GAPDH** |
|  | **IP Flag** |  | **IP IRE1α** |

**Figure S8**

|  | **shNC** | | | **shUPF3B** | | | **shUPF3B+WT** | | | **shUPF3B+RRM** | | | **shUPF3B+EBM** | | |
| --- | --- | --- | --- | --- | --- | --- | --- | --- | --- | --- | --- | --- | --- | --- | --- |
| **ATF3** | **1** | **1** | **1** | **2.680496** | **2.680496** | **2.717914** | **1.58631** | **1.488242** | **1.38631** | **2.437083** | **2.573583** | **2.377912** | **2.609263** | **2.709263** | **2.765751** |
| **PERK** | **1** | **1** | **1** | **1.650047** | **1.459624** | **1.56508** | **1.116455** | **1.261537** | **1.164147** | **1.63497** | **1.815387** | **1.83497** | **1.634945** | **1.894538** | **1.614672** |
| **ATF4** | **1** | **1** | **1** | **1.515828** | **1.449522** | **1.500991** | **1.095397** | **1.018186** | **1.00858** |  | **1.584189** | **1.42367** | **1.48328** | **1.153475** | **1.424091** |
| **IRE1α** | **1** | **1** | **1** | **3.549215** | **3.111262** | **3.176636** | **1.55724** | **1.441047** | **1.458026** | **2.779415** | **2.966852** | **3.313376** | **3.235804** | **3.169168** | **3.270176** |
| **XBP1s** | **1** | **1** | **1** | **3.72567** | **3.381117** | **3.390811** | **1.424185** | **1.424185** | **1.525462** | **2.549121** | **3.099433** | **3.027919** | **3.313376** | **3.093491** | **3.004445** |
| **ATF6** | **1** | **1** | **1** | **1.031436** | **1.169302** | **1.243567** | **1.120109** | **1.173073** | **1.108227** | **1.135804** | **1.158639** | **1.135804** | **1.131144** | **1.114195** | **1.174561** |
| **PRDG1** | **1** | **1** | **1** | **1.277282** | **1.300078** | **1.13325** | **1.061914** | **1.130269** | **1.138131** | **1.061914** | **1.04006** | **1.0693** | **1.084227** | **1.084227** | **1.186463** |
