# Supplementary material for: UPF3B modulates endoplasmic reticulum stress through interaction with inositol-requiring enzyme-1α
Source: Cell Death Dis. 2024 Aug 13;15(8):587. doi: 10.1038/s41419-024-06973-3 (PMC11322666; doi:10.1038/s41419-024-06973-3)
Supplement: Supplementary file 1 — Supplemental figures and tables [file 41419_2024_6973_MOESM1_ESM.docx]

Supplementary Information

**UPF3B modulates endoplasmic reticulum stress through the interaction with inositol-requiring enzyme-1α**

XingSheng Sun, Ruqing Lin, Xinxia Lu, Zhikai Wu, Xueying Qi, Tianqing Jiang, Jun Jiang, Peiqiang Mu, Qingmei Chen, Jikai Wen and Yiqun Deng

Supplemental Data

Figures S1 Cytotoxicity of inhibitors were evaluated in cells. HEK293T cells at 70% confluence were treated with DMSO and various of inhibitors for 1, 2 or 4 h. Cell viability was then determined with CCK-8 (n = 6).

Figures S2 The interaction between UPF3B and IRE1α is not affected by Ribonuclease A

Co-IP analysis of the interaction between IRE1α and UPF3B under RNase A. The cells lysates were added RNase A (10 μg/mL) and subjected to immunoprecipitation with the IRE1α antibody, then visualized by western blotting.

Figure S3 Colocalization analysis of UPF3B and IRE1α (A), UPF3B and ER (B) by image J.

Figure S4 The interaction of UPF3B and IRE1α were detected in shUPF1 and shUPF2 cells. The cell lysates were immunoprecipitated with anti-IRE1α antibody, and the precipitates and whole cell lysates were then analyzed by western blotting. IgG was used as a negative control in IP assay.

Figure S5 The relative expression levels of UPF3B and IRE1α proteins are collected from human proteome map database, which is the web-based resource that reorganizes mass spectrometry-based proteomics data to explore expressed proteins in tissues and cells obtained from human.

Figure S6 (A) UPF3A interacts with IRE1α. Co-IP analysis of the interaction between IRE1α and UPF3A. HEK293T cells were transfected with plasmids encoding the UPF3A-HA and the cell lysates were immunoprecipitated with anti-IRE1α antibody, and the precipitates and whole cell lysates were then analyzed by western blotting. pcDNA3.1 plasmid as the negative control. (B) UPF3A were knocked down stably in HEK293T cells to detect the expression level of target proteins by western blotting. (C) UPF3A was overexpressed in the shUPF3B cell line to detect the expression level of target proteins by western blotting.

Figure S7 The suppression of IRE1α requires the full length of UPF3B.

The levels of phosphorylated IRE1α were evaluated after overexpression of UPF3B mutant plasmid in HEK293T cell line, cells were collected and performed with above antibody for western blotting analysis.

Figure S8 The expression levels of NMD substrates were examined in the presence of complementary UPF3B^WT^ and two truncation mutants in the UPF3B knockdown cell line. Quantitative data are expressed as the mean ± standard error of the mean (SEM) (n=3). **p*<0.05.

Figure S9 The strength of the interaction between IRE1α and UPF3B was negatively correlated with the phosphorylation level of IRE1α. Image J software was using to calculated the grayscale. (A) The data were from figure 4E. Statistical analysis of the phosphorylation level of IRE1α was compared to the control and the interaction efficiency was compared to the IRE1α^WT^ (B) The data were from figure 4F and 4G. Quantitative data are expressed as the mean ± standard error of the mean (SEM). Statistical analysis of the phosphorylation level of IRE1α was compared to the control and the interaction efficiency was compared to the IRE1α^WT^, ****p*<0.001.

Figure S10 BiP inhibits the activation of IRE1α in UPF3B knockdown cell lines

(A) Overexpression of BiP plasmid in shUPF3B cell lines, cells were harvested and subjected to western blotting analysis. (B) Phosphorylation of IRE1α was inhibited when BiP is supplemented in shUPF3B cell line. The data were from figure 5C and S9A. **p*<0.05, ***p*<0.01, ****p*<0.001. (C) The levels of phosphorylated IRE1α were evaluated after siBiP in UPF3B knockdown cell lines. (D) The levels of phosphorylated IRE1α were evaluated after overexpression of UPF3B in BiP knockdown cell lines.

Figure S11 The interaction between IRE1α and UPF3B mutants

Co-IP analysis of the interaction between IRE1α and UPF3B mutants. HEK293T cells were transfected with the Flag tagged UPF3B mutant for 24 h. The cell lysates were subjected to immunoprecipitation with the indicated antibodies, and visualized by western blotting.

Figure S12 The inhibition of IRE1α clusters by overexpression of UPF3B^Y160D^ mutation was attenuated during ER stress

(A) UPF3B mutants were transfected for 24 h in UPF3B knockdown cell lines and treated with corresponding Tm or Tg for 3 h. The cells were stained with Hoechst 33342. The scale is 10 μm. (B) Percentage of total cell fluorescence intensity found in clusters at each time point. (C) Distribution of cluster sizes during the time course. (D) Distribution of cluster fluorescence intensities at each time point. Each dot represents 1 field, 5 fields were analyzed for each condition and an average of 60 cells were analyzed per condition in each experiment. The results are the means ± SEMs of at three independent experiments. Statistical significance was defined as **p*<0.05, ***p*<0.01 or ****p*<0.001.

Figure S13 (A) The strength of the interaction between IRE1α and UPF3B were negatively correlated with the phosphorylation level of IRE1α. The data were from figure 7A and 7G-7I, Image J software was using to calculated the grayscale. Statistical analysis of the phosphorylation level of IRE1α was compared to the control and the interaction was compared to the UPF3B^WT^. (B) The strength of the interaction between UPF2 and UPF3B. The data were from figure 7J-7L. The results are the means ± SEMs of at three independent experiments. Statistical significance was defined as ****p*<0.001.

Figure S14 Flow cytometry analysis of apoptotic changes by supplemented with different UPF3B mutants under Tg (2 μM for 3 h) treatment in shUPF3B cell lines. Data statistical analyses were performed on Annexin-V+/PI+ double positive cells. The results are the means ± SEMs of at three independent experiments. Statistical significance was defined as ****p*<0.001.

Table S1 The primer sequences applied in current study

| qPCR primers | Forword | Reverse |
| --- | --- | --- |
| PERK | GAGCAGATTCATGGAAACAGC | GTTAAGGTCCTGACTCTCTCC |
| IRE1α | TGCAGGTCCCAACACATGTGG | TCAGGCCTTCATTATTCTTGC |
| ATF6 | TCGGTCAGTGGACTCTTATT | CCAGTGACAGGCTTATCTTC |
| XBP1s | AAGAACACGCTTGGGAATGG | CTGCACCTGCTGCGGAC |
| ATF3 | GAGGCGAGCAGAAAGAAATAAG | GTAAGGCTAGAAGGCACTCAC |
| ATF4 | GTCAGTCCCTCCAACAACAGC | GTCATCTATACCCAACAGGGC |
| PRDG1 | TGCGCCTCTTACCATATGAC | GGCAGTTCATACTGGGACCT |
| GAPDH | CTTTTGCGTCGCCAG | TTGATGGCAACAATATCCAC |

Table S2 The antibodies applied in current study

| Antibodies | Species | Catalog | Manufacturer |
| --- | --- | --- | --- |
| IRE1α | Rabbit | 14C10 | Cell Signal Technology (Beverly, MA, USA) |
| IRE1α | Mouse | sc-390960 | Santa Cruz Biotechnology (CA, USA) |
| UPF3B | Rabbit | Ab134566 | Abcam (London, UK) |
| UPF3B | Mouse | sc-514564 | Santa Cruz Biotechnology (CA, USA) |
| UPF1 | Rabbit | D161327 | Sangon Biotech (Shanghai, China) |
| UPF2 | Rabbit | A7091 | ABclonal (Wuhan, China) |
| UPF3A | Rabbit | PA5-110551 | Thermo Fisher Scientific (MA, USA) |
| PERK | Rabbit | AF6771 | Beyotime Biotech (Shanghai, China) |
| p-PERK | Rabbit | AF5902 | Beyotime Biotech (Shanghai, China) |
| p-IRE1α | Rabbit | #13013 | Signalway Antibody (MD, USA) |
| ATF4 | Rabbit | ET1612-37 | Huabio (Hangzhou, China) |
| ATF6 | Rabbit | D262665 | Sangon Biotech (Shanghai, China) |
| XBP1s | Rabbit | A17007 | ABclonal (Wuhan, China) |
| p-eIF2α | Rabbit | AF1237 | Beyotime Biotech (Shanghai, China) |
| CHOP | Mouse | L63F7 | Cell Signal Technology (Beverly, MA, USA) |
| BiP | Rabbit | C50B12 | Cell Signal Technology (Beverly, MA, USA) |
| Phosphoserine/threonine | Rabbit | PP2551 | ECM biosciences (Kentucky, USA) |
| Flag | Rabbit | F7425 | Sigma Aldrich (St. Louis, MO, USA) |
| Myc | Rabbit | #2278 | Cell Signal Technology (Beverly, MA, USA) |
| HA | Rabbit | #3724 | Cell Signal Technology (Beverly, MA, USA) |
| GAPDH | Mouse | sc-32233 | Santa Cruz Biotechnology (CA, USA) |
| Goat anti-Mouse IgG H&L (Alex Fluor 594) | Mouse | ab150117 | Abcam (London, UK) |
| Goat anti-Rabbit IgG H&L (Alex Fluor 488) | Rabbit | ab150079 | Abcam (London, UK) |
| HRP-linked anti-Mouse IgG | Mouse | #3724 | Cell Signal Technology (Beverly, MA, USA) |
| HRP-linked anti-Rabbit IgG | Rabbit | #7074 | Cell Signal Technology (Beverly, MA, USA) |
